# The crystal structures of *Thermus thermophilus* CMP kinase complexed with a phosphoryl group acceptor and donor

**DOI:** 10.1371/journal.pone.0233689

**Published:** 2020-05-29

**Authors:** Ryosuke Mega, Noriko Nakagawa, Seiki Kuramitsu, Ryoji Masui

**Affiliations:** 1 Graduate School of Frontier Biosciences, Osaka University, Suita, Osaka, Japan; 2 Arid Land Research Center, Tottori University, Tottori, Japan; 3 Department of Biological Sciences, Graduate School of Sciences, Osaka University, Toyonaka, Osaka, Japan; 4 Graduate School of Science, Osaka City University, Osaka, Japan; George Washington University, UNITED STATES

## Abstract

Nucleoside monophosphate kinases play crucial roles in biosynthesis and regeneration of nucleotides. These are bi-substrate enzymes that catalyze reversible transfers of a phosphoryl group between ATP and nucleoside monophosphate. These enzymes are comprised of the CORE domain, the NMP-binding domain, and the LID domain. Large conformational rearrangement of the three domains occurs during the catalytic cycle. Although many structures of CMP kinase have been determined, only limited structural information has been available on the conformational changes along the reaction pathway. We determined five crystal structures of CMP kinase of *Thermus thermophilus* HB8 in ligand-free form and the CMP "open", CMP "closed", ADP-CDP-Gd^3+^-, and CDP-bound forms at resolutions of 1.7, 2.2, 1.5, 1.6, and 1.7 Å, respectively. The ligand-free form was in an open conformation, whereas the structures of the CMP "closed", ADP-CDP-Gd^3+^-, and CDP-bound forms were in a closed conformation, in which the shift of the NMP-binding domain and LID domain caused closure of the substrate-binding cleft. Interestingly, the CMP "open" form was in an open conformation even with CMP bound, implying intrinsic conformational fluctuation. The structure of the ADP-CDP complex is the first structure of CMP kinase with a phosphoryl group donor and an acceptor. Upon simultaneous binding of ADP and CDP, the side chains of several residues in the LID domain moved toward the nucleotides without global open−closed conformational changes compared to those in the CMP "closed" and CDP complexes. These global and local conformational changes may be crucial for the substrate recognition and catalysis. The terminal phosphate groups of ADP and CDP had similar geometry to those of two ADP in AMP kinase, suggesting common catalytic mechanisms to other nucleoside monophosphate kinases. Our findings are expected to contribute to detailed understanding of the reaction mechanism of CMP kinase.

## Introduction

Nucleoside monophosphate (NMP) kinases are key enzymes in the biosynthesis and regeneration of ribo- and deoxyribonucleoside triphosphates, which are the building blocks of RNA and DNA, respectively [1]. NMP kinases include adenylate kinase (AMPK), cytidylate kinase (CMPK), guanylate kinase, and thymidylate kinase, which catalyze conversions of respective NMPs to the corresponding nucleoside diphosphates. NMP kinases are bi-substrate enzymes that bind both substrates simultaneously to catalyze phosphoryl transfers.

The structures of these enzymes consist of three major domains termed CORE domain, NMP-binding domain, and LID domain [2]. The CORE domain contains the phosphate-binding loop (P-loop) as a common motif found in most ATP-binding proteins. During the phosphotransfer reaction, there are significant rearrangements of the three domains, which are mainly attributed to rigid-body movements of the LID and NMP-binding domains with respect to the CORE domain [2]. Many structures of NMP kinases, in particular AMPK, in different conformational states have been captured in crystallography and NMR experiments [3–7]. Typically, AMPK adopts an open conformation in the absence of ligand. In the ligand-bound state, the LID and NMP-binding domains close over substrates, leading to a closed conformation. However, recent studies suggested the existence of a ligand-bound open state and a ligand-free closed state, implying that ligand binding would precede the domain closure [6,8,9]. Many studies have attempted to explain how AMPK could transition from an unbound conformation to a bound conformation in complex with a ligand [6,8,10–15]. Two popular models for AMPK-ligand binding have been proposed: the induced fit and conformational selection mechanisms [16–18]. More complex mechanisms have also been proposed, but these have remained controversial.

Several crystal structures of CMPK from *Escherichia coli* and other organisms are known: ligand-free forms from *E*. *coli* [19], *Mycobacterium abscessus* [20], *Mycobacterium smegmatis* [20], and *Yersinia pseudotuberculosis* [21]; CMP-bound forms from *E*. *coli* [22], *M*. *abscessus* [20], and *Staphylococcus aureus* [23]; and CDP-bound form from *E*. *coli* [19]. The crystal structures of *E*. *coli* CMPK complexed with CMP analogues have also been determined [22]. In addition, the NMR solution structure of the ligand-free (apo) form of CMPK from *Streptococcus pneumoniae* has been reported [24]. The structural organizations of the three domains in CMPKs are fundamentally the same as AMPKs. Unlike AMPK, the *E*. *coli* CMPK undergoes dynamic conformational change only in the LID domain by CMP binding [22]. Comparison of the ligand-free form with the CDP-bound form also revealed movement of the NMP-binding domain upon CDP binding [19]. It is uncertain whether these apparent differences in domain movement upon ligand binding between AMPK and CMPK are fundamental. Actually, an NMR study of the solution structure of *S*. *pneumoniae* CMPK reported the LID domain was greatly fluctuated in the ligand-free state [24]. More structural information is required to fully understand how ligand binding is coupled with conformational change in the binding site of CMPK. In particular, structural information of CMPK complexed with a phosphoryl group donor is lacking.

In this research, we determined structures of the ligand-free-, CMP-bound, CDP-bound, and ADP-CDP-bound forms of CMPK from an extremely thermophilic eubacterium, *Thermus thermophilus* HB8 (ttCMPK). We first succeeded in determining the tertiary complex of CMPK with ADP and CDP. Furthermore, we determined the structures of ttCMPK with CMP not only in a closed conformation but also in an open conformation. Based on these structures, we elucidated the detailed conformational changes upon binding of ligands in the reaction pathway of CMPK.

## Materials and methods

### Protein overexpression and purification

The expression plasmid of ttCMPK (TTHA0458) was transformed into *E*. *coli* BL21(DE3). This plasmid was constructed by ligating the amplified *ttha0458* fragment into the NdeI and BamHI sites of pET-11a (Novagen) and provided from RIKEN BioResource Center. The transformant was cultured at 310 K in Luria-Bertani medium containing 1 mM ampicillin for 20 h. The protein was overexpressed without induction by IPTG probably due to the basal expression of T7 RNA polymerase from the *lacUV5* promoter even in the absence of inducer. The cells were harvested by centrifugation. The cells (22 g) were ultrasonicated in 20 mM Tris-HCl, pH 8.0, 5 mM 2-mercaptoethanol and 50 mM NaCl, and the lysate was incubated at 343 K for 10 min. After centrifugation, proteins in the supernatant were precipitated by 1.5 M ammonium sulfate. The collected precipitate was suspended in 50 mM Tris-HCl, pH 9.0. After desalting, the protein solution was purified by successive chromatographic steps with RESOURCE Q, HiTrap Heparin and HiLoad 16/60 Superdex 75 pg columns (GE Healthcare). The purified protein (9.3 mg/mL) was stored in 50 mM Tris-HCl, pH 9.0 and 150 mM NaCl.

To obtain the selenomethionine-labelled (SeMet) protein, *E*. *coli* B834(DE3) cells (Novagen) transformed with the expression plasmid were grown at 310 K for 24 h in LeMaster medium [25] containing 1 mM ampicillin and 10 g/L lactose, instead of glucose, and harvested by centrifugation. SeMet protein was purified in a similar manner to native protein.

### Crystallization, data collection, and determination

The initial screening and optimization of crystallization conditions were performed using the hanging-drop vapor-diffusion method at 293 K. The crystals of free enzyme were obtained by mixing 2 μL native protein (9.5 mg/mL), 0.4 μL of 0.1 M NAD^+^, and 1.6 μL reservoir solution containing 60 mM sodium acetate, pH 4.5, 40% ethylene glycol, and 15% PEG1000. The crystals of the open form complex with CMP were obtained by mixing 2 μL SeMet protein, 2 μL of 10 mM CMP and 10 mM MgCl_2_, and 2 μL reservoir solution containing 0.6 M sodium succinate, pH 7.0. The concentration of CMP (10 mM) was higher compared to the *K*_m_ value (10 μM) of ttCMPK. The crystals were soaked for a few minutes in reservoir solution containing 20% glycerol. The crystals of the closed form complexed with CMP were obtained by mixing 2 μL native protein, 2 μL of 10 mM CMP, 10 mM β,γ-methyleneadenosine 5’-triphosphate (AMP-PCP), and 10 mM MgCl_2_ and 2 μL reservoir solution containing 100 mM Tris-HCl, pH 8.0, and 2.4 M sodium formate. The crystals were soaked for a few minutes in reservoir solution containing 20% glycerol. The crystals of closed form complex with CDP were obtained by mixing 2 μL native protein, 2 μL of 10 mM CMP and 10 mM ATP, and 2 μL reservoir solution containing 100 mM Tris-HCl, pH 8.0, 2.6 M sodium formate, and 20% glycerol. The crystals of closed form complex with CDP, ADP and GdCl_3_ were obtained by mixing 2 μL native protein, 2 μL of 10 mM CMP, and reservoir solution containing 100 mM Tris-HCl, pH 8.4, and 2.1 M sodium formate. The crystals were soaked for 110 min in reservoir solution containing 10 mM CMP, 10 mM ATP, 10 mM GdCl_3_, and 20% glycerol. Crystals were flash-cooled to 90 K, and diffraction data were collected at the RIKEN Structural Biology Beamline I BL45XU-PX [26] at SPring-8 (Hyogo, Japan). The diffraction data sets for the SeMet protein crystal were collected at the selenium peak (0.9791 Å) at 90 K at the BL45PX station at SPring-8. Data were processed using the HKL2000 program suite [27].

The structure of open form complex with CMP was determined by the single-wavelength anomalous dispersion method using the program SOLVE/RESOLVE [28]. Two Se sites (out of three possible sites) were identified. The structure of the free enzyme was determined by molecular replacement with the program MOLREP [29] in CCP4 [30], using the structure of open form complex with CMP as a starting model. The structures of the other closed form complexes were solved using *E*. *coli* CMP kinase complexed with CDP (PDB code 2CMK) [19] as a starting model for molecular replacement, using the program MOLREP. The automatic tracing procedure in the program ARP/wARP [31] was utilized to build the initial model. The remainder of the molecule was built into the electron density map using the programs MIFit [32] or Coot [33], and model refinement was performed with the program CNS [34]. All ligands were placed by using the program PHENIX [35] or ARP/wARP. The ratios of the final model residues in the most-favored region and disallowed region of the Ramachandran plot were calculated by MolProbity [36]. Data-collection statistics and processed data statistics are presented in Table 1. Least squares comparisons between two structures were carried out using the program LSQKAB in CCP4 [30]. Hydrogen bonds were calculated using the program Chimera [37]. Structure diagrams were drawn using the program PyMOL (https://pymol.org/2/).

**Table 1:** Data collection and refinement statistics.

Data set	Ligand free	CMP	CMP	CDP	ADP-CDP-Gd^3+^
	open form	open form	closed form	closed form	closed form
PDB code	(3W90)	(3W8N)	(3AKE)	(3AKD)	(3AKC)
**Data collection**					
Space group	*P*2_1_2_1_2_1_	*P*3_1_	*P*6_5_	*P*6_5_	*P*6_5_
Unit cell					
a, b, c (Å)	a = 35.7	a = 49.2	a = 61.7	a = 61.9	a = 62.2
	b = 48.4	b = 49.2	b = 61.7	b = 61.9	b = 62.2
	c = 110.0	c = 98.1	c = 111.3	c = 111.1	c = 111.0
α, β, γ (°)	90, 90, 90	90, 90, 120	90, 90, 120	90, 90, 120	90, 90, 120
Resolution (Å) ^a^	50.00–1.60	50.00–2.20	50.00–1.50	50.00–1.60	50.00–1.65
	(1.66–1.60)	(2.28–2.20)	(1.55–1.50)	(1.66–1.60)	(1.71–1.65)
Unique reflections ^a^	26,020	26,811	38,410	29,707	28,624
	(2,549)	(2,684)	(3,801)	(1,768)	(2,453)
Completeness (%) ^a^	99.9 (99.6)	99.5 (99.2)	99.9 (99.8)	93.1 (55.7)	97.7 (84.4)
Redundancy ^a^	6.6 (4.7)	2.0 (1.9)	8.1 (7.8)	11.1 (7.7)	9.2 (6.8)
Average *I/σ(I)* ^a^	28.4 (6.9)	23.3 (3.7)	45.7 (9.2)	53.0 (3.4)	38.2 (3.4)
*R*_merge_ (%) ^a^^,^^b^	6.2 (27.7)	4.9 (26.2)	6.6 (34.3)	3.8 (26.2)	6.7 (28.7)
**Refinement**					
Resolution (Å) ^a^	44.29–1.65	42.62–2.20	38.56–1.50	38.58–1.60	38.65–1.65
	(1.75–1.65)	(2.34–2.20)	(1.59–1.50)	(1.70–1.60)	(1.75–1.65)
No. of reflections ^a^	23,431	26,009	38,329	29,665	28,585
	(3,383)	(3,632)	(5,677)	(3,184)	(3,936)
*R*_work_ (%) ^a^^,^^c^	21.4 (23.4)	19.9 (26.4)	20.6 (23.1)	21.0 (25.5)	19.9 (26.0)
*R*_free_ (%) ^a^^,^^d^	24.5 (27.9)	24.5 (31.9)	22.9 (25.2)	23.6 (30.5)	22.0 (28.9)
No. of atoms					
Protein	1506	1518	1577	1540	1591
Ligand/Ion	0	21	21	25	53
Water	170	84	135	103	116
Average B factor (Å^2^)	21.5	37.6	19.5	22.3	21.4
Protein	20.2	37.4	19.0	21.6	20.7
Ligand Root mean square deviation		35.3	16.4	21.0	28.6
Bond lengths (Å)	0.020	0.008	0.019	0.034	0.019
Bond angles (°)	1.9	1.4	2.1	2.6	2.0
Ramachandran plot					
Favored regions (%) ^e^	98.4	98.0	99.0	98.0	98.5
	(189/192)	(193/197)	(200/202)	(194/198)	(203/206)
Allowed regions (%) ^e^	1.6 (3/192)	2.0 (4/197)	1.0 (2/202)	2.0 (4/198)	1.0 (2/206)
Disallowed regions (%) ^e^	0.0	0.0	0.0	0.0	0.0

^a^ The values in parentheses are for the outermost shell.

^b^ *R*_merge_ = Σ_*h*_Σ_*i*_ |*I*(*h*,*i*) − <*I*(*h*)>|/Σ_*h*_Σ_*i*_ *I*(*h*,*i*) where *I*(*h*,*i*) is the intensity value of the *i*th measurement of *h*, and <*I*(*h*)> is the corresponding mean value of *I*(*h*) for all *i* measurements.

^c^ *R*_work_ = Σ||*F*_obs_| − |*F*_calc_||/Σ|*F*_obs_|, where |*F*_obs_| and |*F*_calc_| are the observed and calculated structure factor amplitudes, respectively.

^d^ *R*_free_ is the same as *R*_work_ but calculated with a 10% subset of all reflections that were never used in crystallographic refinement.

^e^ The value was calculated using MolProbity [36]. The values in parentheses are the numbers of residues.

The coordinates and structure factors of ttCMPK and its complexes with nucleotides were deposited in the Protein Data Bank with the following accession codes: 3W90 (ligand free), 3W8N (CMP "open" complex), 3AKE (CMP "closed" complex), 3AKD (CDP complex), and 3AKC (ADP-CDP-Gd^3+^ complex).

## Results

### Overall structures

**Fig 1:**
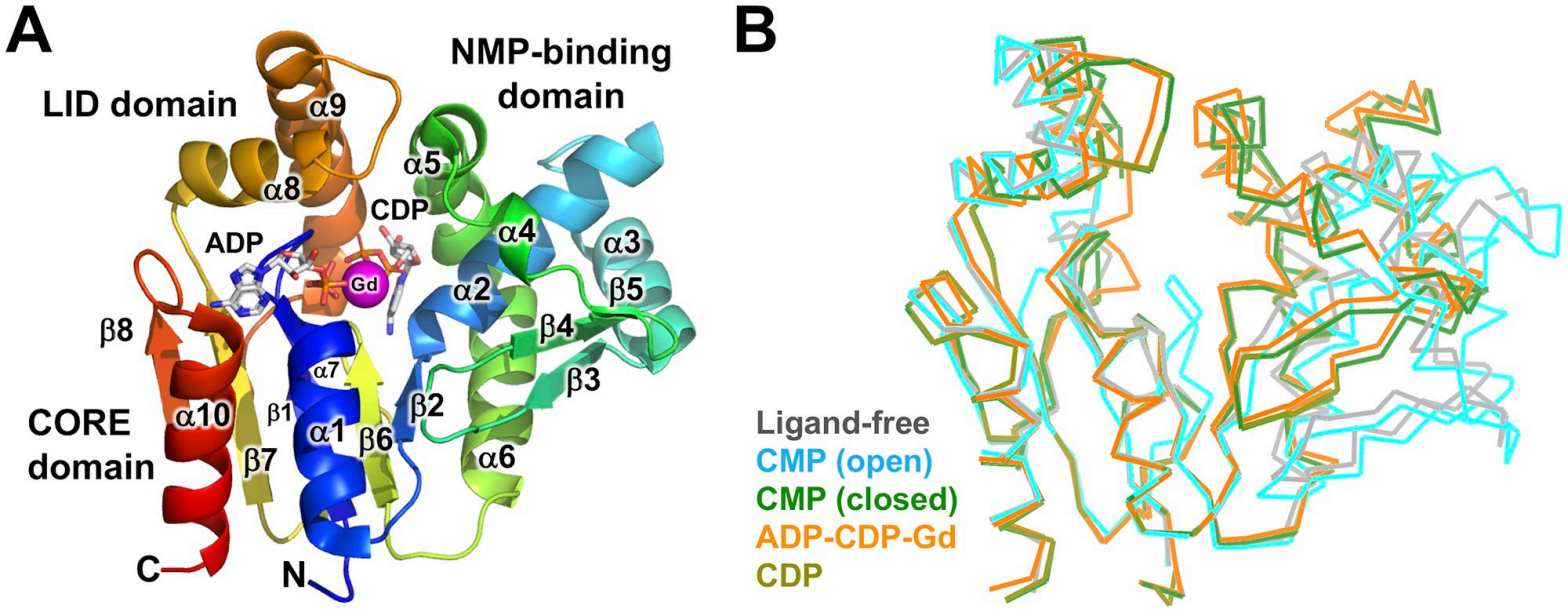
Crystal structure of ttCMPK. (A) Overall structure of ttCMPK in complex with ADP, CDP, and Gd^3+^. The structure is shown in a schematic representation, colored in a spectrum from the N-terminus (blue) to the C-terminus (red). The bound nucleotides are shown in sticks. (B) Superimposition of the overall structure of ligand-free form (gray; PDB code 3W90), CMP "open" complex (cyan; 3W8N), CMP "closed" complex (green; 3AKE), ADP-CDP-Gd^3+^ complex (orange; 3AKC), and CDP complex (olive; 3AKD).

**Fig 2:**
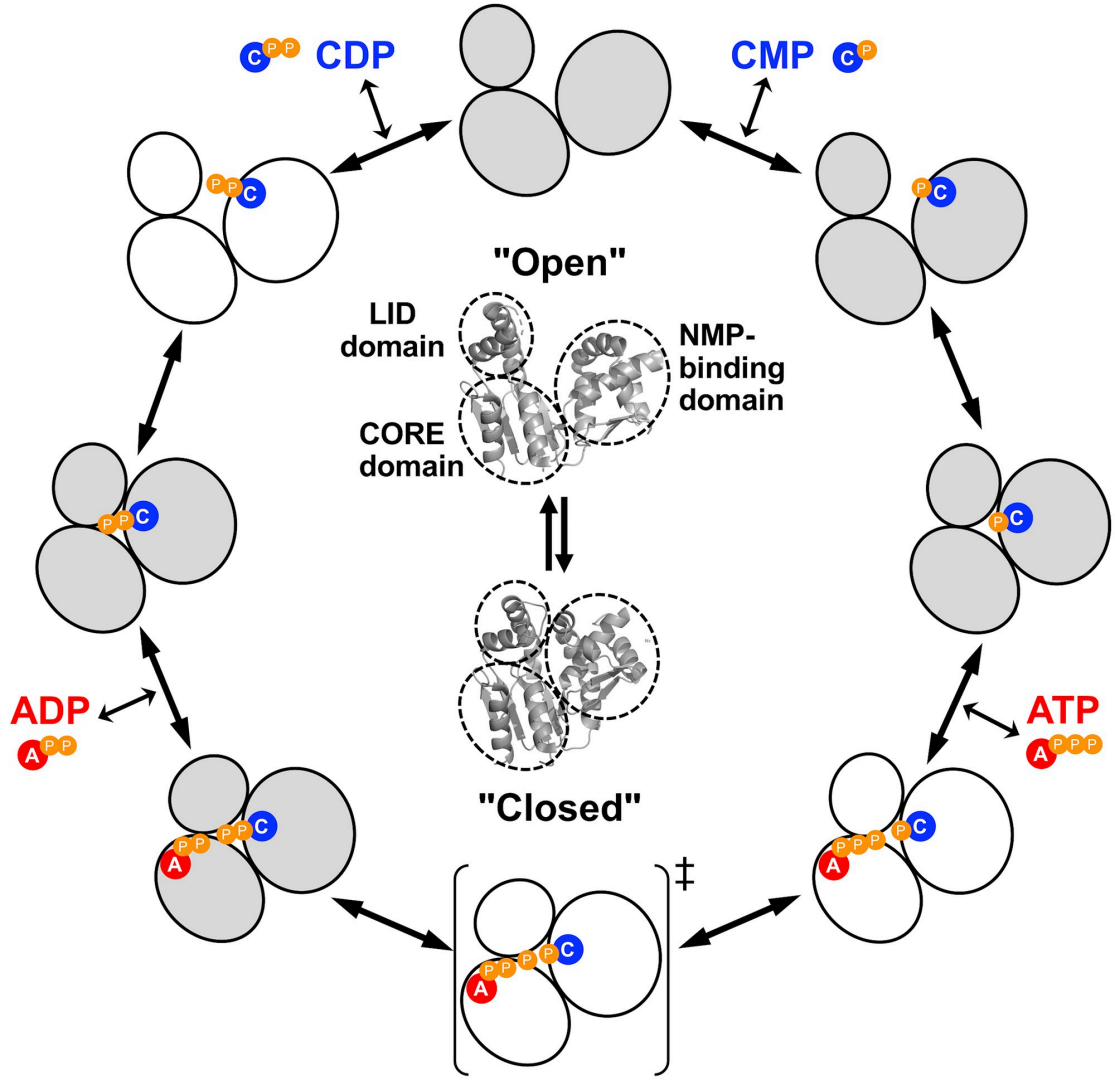
A schematic representation of the reaction cycle of ttCMPK. The series of the conformational changes which were determined in this study (gray) or hypothesized (white) to occur upon substrate binding are shown. “A”, “C”, and “P” represent adenosine, cytidine, and phosphate moieties, respectively. The symbol ‡ indicates the transition state of phosphoryl transfer.

We determined five crystal structures of ttCMPK in ligand-free form, the CMP-bound "open" form, CMP-bound "closed" form, ADP-CDP-Gd^3+^-, and CDP-bound forms at resolutions of 1.7, 2.2, 1.5, 1.6, and 1.7 Å, respectively (Table 1, Fig 1). Structural differences between the two CMP-bound forms will be explained in later sections. The structure of the ADP-CDP-Gd^3+^ complex is the first structure of CMPK with two nucleotides, a phosphoryl group donor, and an acceptor. The overall structure of ttCMPK consists of three domains: CORE domain, NMP-binding domain, and LID domain (Figs 1 and 2). Such a domain organization is essentially the same as those of *E*. *coli* and *S*. *aureus* CMPKs [19,22,23].

When the five determined structures were structurally aligned based on superimposing the backbone structure of their CORE domains, it was evident the ligand-free form and CMP "open" complex exist in open conformation compared to the other three (Fig 1B). In each of the latter three complexes, the shift of the NMP-binding domain and LID domain caused closure of the substrate-binding cleft, leading to closed conformation.

In the ligand-free form, residues 1–2, 50–54, 157–158, and 209 are missing from the model due to insufficient electron density. Similarly, in the CMP "open" complex, residues 1–2, 154–158, and 208–209 are missing. These two structures are open conformation, as described later. In the CMP "closed" and CDP complexes, residues 176–177 and 173–178 are missing, respectively, whereas the ADP-CDP-Gd^3+^ complex has no missing residues. These three complexes are closed conformation. Residues 154–158, in the loop region in the LID domain, were not disordered in these three structures, suggesting that this region formed a rigid structure by domain closure. In contrast, in the structures of the CMP "closed" and CDP complexes, residues 173–178 at the bottom of the LID domain were disordered, probably due to the hinge motion of this domain.

### Structures of the ligand-free form and CMP "open" complex

The reaction catalyzed by CMPK is thought to follow a random bi-bi mechanism by analogy with AMPK [38]. Although there are four different orders of substrate binding and product release, we assumed a scheme of substrate binding to ttCMPK shown in Fig 2. In the following, we describe in detail the determined structures of ttCMPK along this reaction cycle: the ligand-free form, CMP "open" complex, CMP "closed" complex, ADP-CDP-Gd^3+^ complex, and CDP complex.

**Fig 3:**
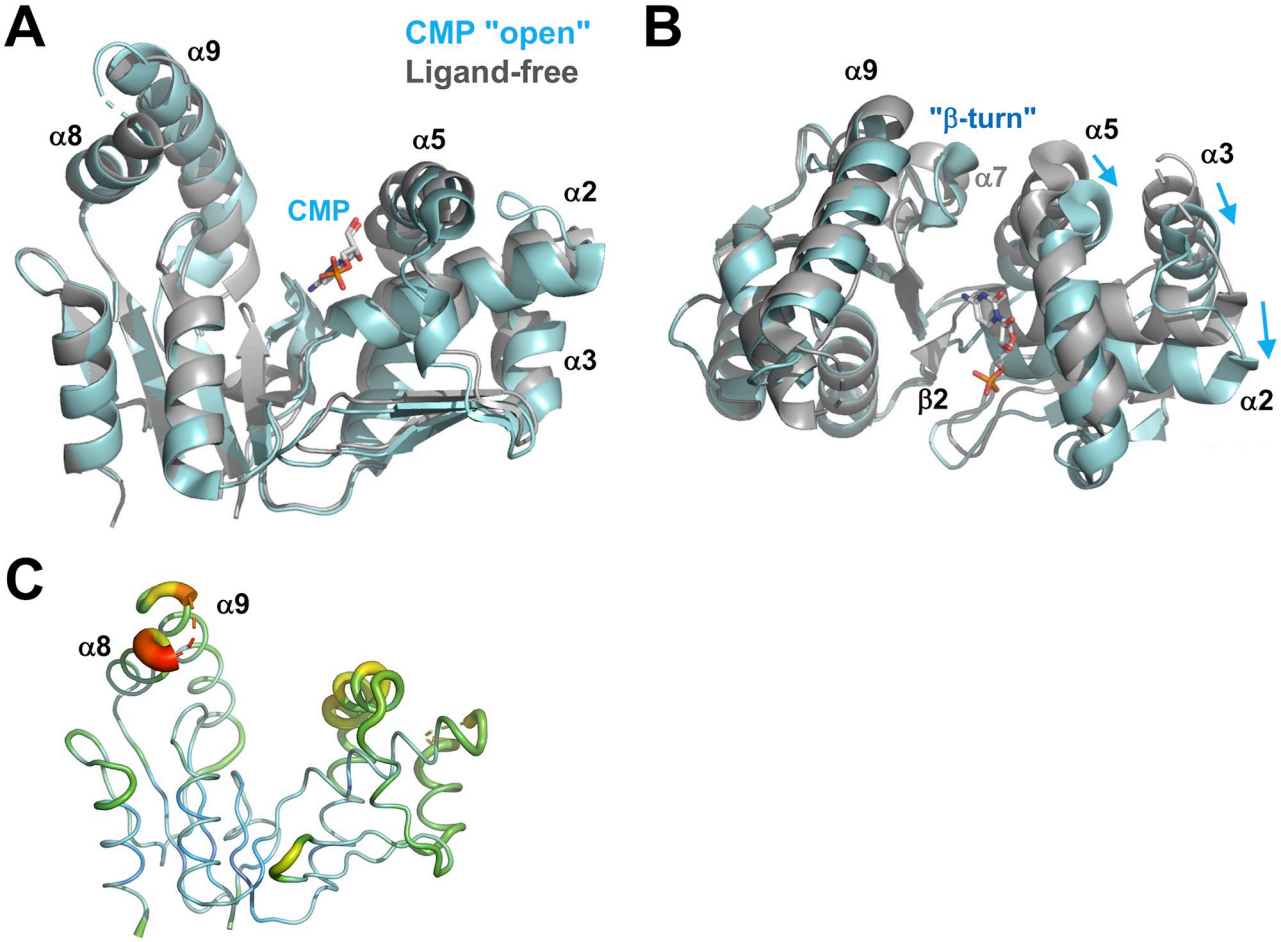
Superimposition of the structures of the ligand-free form (gray) and the CMP "open" complex (cyan). (A) The two structures are superimposed on the basis of C_α_ positions in the CORE domain. CMP is shown as sticks colored according to atom type. Disordered regions are shown as dotted lines. (B) Compared to (A), this figure is rotated by 90° around the horizontal axis. (C) B-factor diagram of the ligand-free form represented by the B-factor putty program in PyMOL. The B-factor values are illustrated by color, ranging from low (blue) to high (red). The average B factor is 21.5 Å^2^.

When the structure of the CMP-bound form, which was cocrystallized with CMP, was determined, an unexpected finding was that this structure was in open conformations that resembled that of the ligand-free form (Fig 3). The root mean square deviation (RMSD) value between these two structures (193 C_α_ atoms) was 3.09 Å. However, there are significant differences between them. The most striking difference is a shift in the position of the NMP-binding domain relative to the CORE domain (Fig 3). As the conformations of the NMP-binding domain are almost the same in the two forms, this shift can be described by rotation or swivel of this domain (Fig 3B). The region around the β2 strand appears to be the hinge or pivot point of this rotation. One of the farthest points from β2 is the C-terminal end of the α2 helix, at which the difference between the two is 5.8 Å. Similarly, the difference in the N-terminal end of the α3 helix is 5.6 Å. However, it should be noted that the observed shift of the NMP-binding domain does not lead to closure of the cleft (Fig 3A). The direction of this shift appears to be perpendicular to that of the domain closure found in the other ligand-bound forms. Therefore, it can be described that the CMP complex exists in an open conformation. In the following sections, we designate this complex as the CMP "open" complex.

In the CORE domain, the regions between β6 and β7 have different conformations. In the ligand-free form, this region forms a short α-helix (α7), whereas the corresponding region forms a β-turn in the CMP "open" complex (Fig 3). The β-turn in this region is found only in the CMP "open"complex among the five determined structures.

Also in the LID domain, the N- and C-terminal ends of the α9 helix have different conformations (Fig 3A). In the CMP "open" complex, the α9 helix shows significantly higher B factors (temperature factors) (average, 58.1 Å^2^) than the rest of the molecule (average, 34.6 Å^2^) (Fig 3C), and is partially disordered, suggesting that this region is flexible. Importantly, in the CMP "open" complex where the substrate-binding cleft is in an open conformation, CMP bound to the NMP-binding domain in a similar way to that in the CMP "closed" complex where the cleft is closed, as discussed later.

### Structure of the CMP "closed" complex

**Fig 4:**
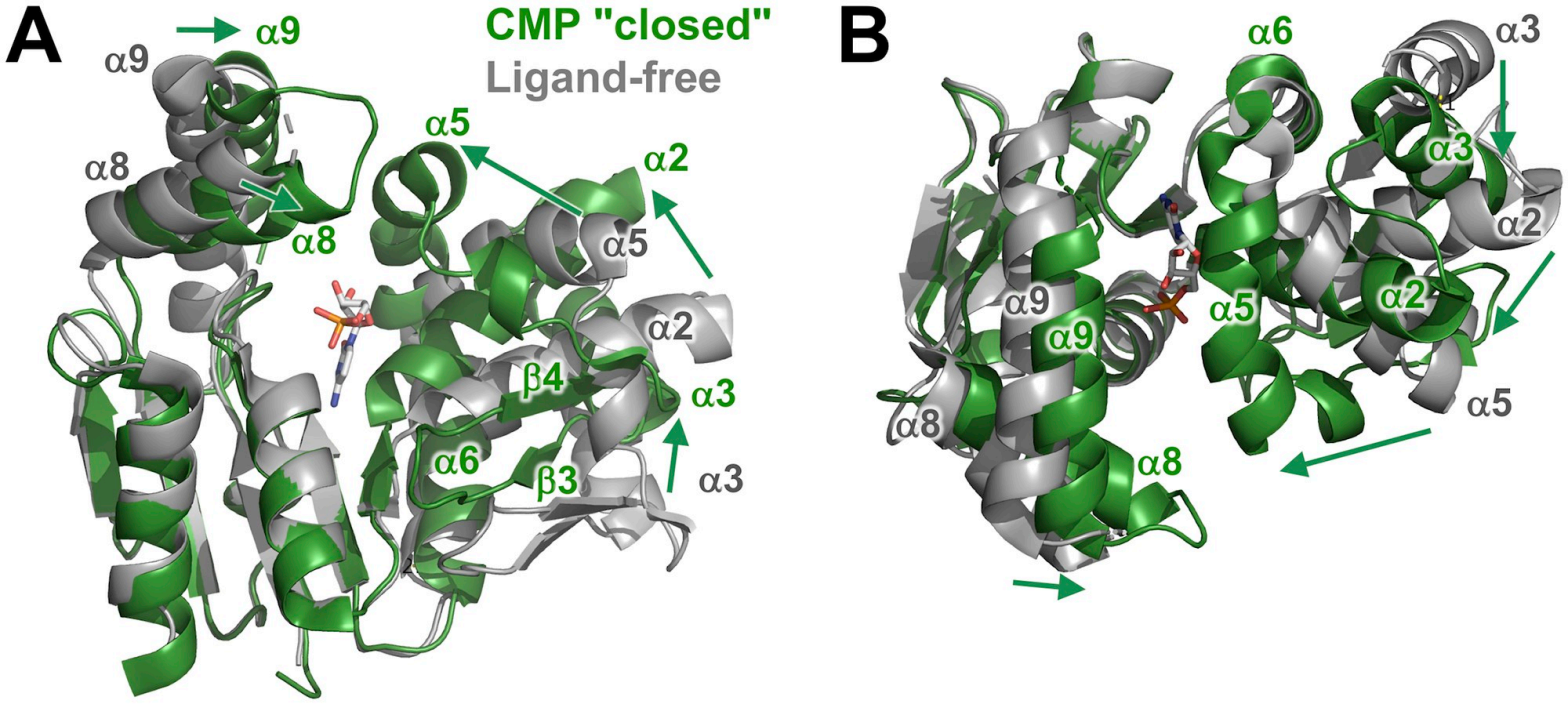
Structural comparison of the CMP "closed" complex (green) with the ligand-free form (gray). (A) The structures are superimposed on the basis of C_α_ positions in the CORE domain. CMP is shown as sticks colored according to atom type. Disordered regions are shown as dotted lines. (B) Compared to (A), this figure is rotated by 125° around the horizontal axis.

We also solved another structure of a CMP-bound form, which was crystallized under a different condition. In contrast to the CMP "open" complex, this complex showed a closed conformation. We designate this complex as the CMP "closed" complex. Comparison of the overall structure of the CMP "closed" complex with the ligand-free form (Fig 4) revealed large shifts in the position of the NMP-binding domain and LID domain relative to the CORE domain. The RMSD value between these two structures (196 C_α_ atoms) was 6.56 Å. These shifts lead to closure of the substrate-binding cleft, where CMP is bound. It should be mentioned here that crystallization condition for the CMP "closed" complex contained AMP-PCP, an ATP analogue. No electron density of AMP-PCP was found, and no significant movement of the side chains was observed in the ATP-binding site. Although these might be probably due to weak binding and low occupancy, even a weak binding ligand in the ATP-binding site might stabilize a closed conformation.

The most striking change in the conformation was found in the NMP-binding domain. The differences in the positions of the C-terminal end of the α2 helix, the C-terminal end of the α3 helix, and the N-terminal end of the α5 helix are 10.8, 11.4, and 13.0 Å, respectively (Fig 4A). Similarly, those of the β3 and β4 strands are approximately 7.0 and 8.0 Å, respectively. As a result, it appears the NMP-binding domain moves in toward CMP in the CMP "closed" complex.

However, the α6 helix shows less shift (< 2 Å) between the two forms (Fig 4B). The C-terminal end of the α5 helix, which is followed by the α6 helix, shows no significant shift; the N-terminal end of the α3 helix, which is near the α6 helix, also shows less shift (~4 Å), although this region of the α3 is disordered (Fig 4B). Therefore, it can be indicated that the closure of the cleft occurs by rotation of the NMP-binding domain around the α6 helix as an axis.

In the LID domain, the α8 and α9 helices move in toward the cleft (Fig 4). The loop region between these helices shows ordered structure in the CMP "closed" complex, in contrast to that in the ligand-free form. These also contribute to changes from an open to a closed conformation.

**Fig 5:**
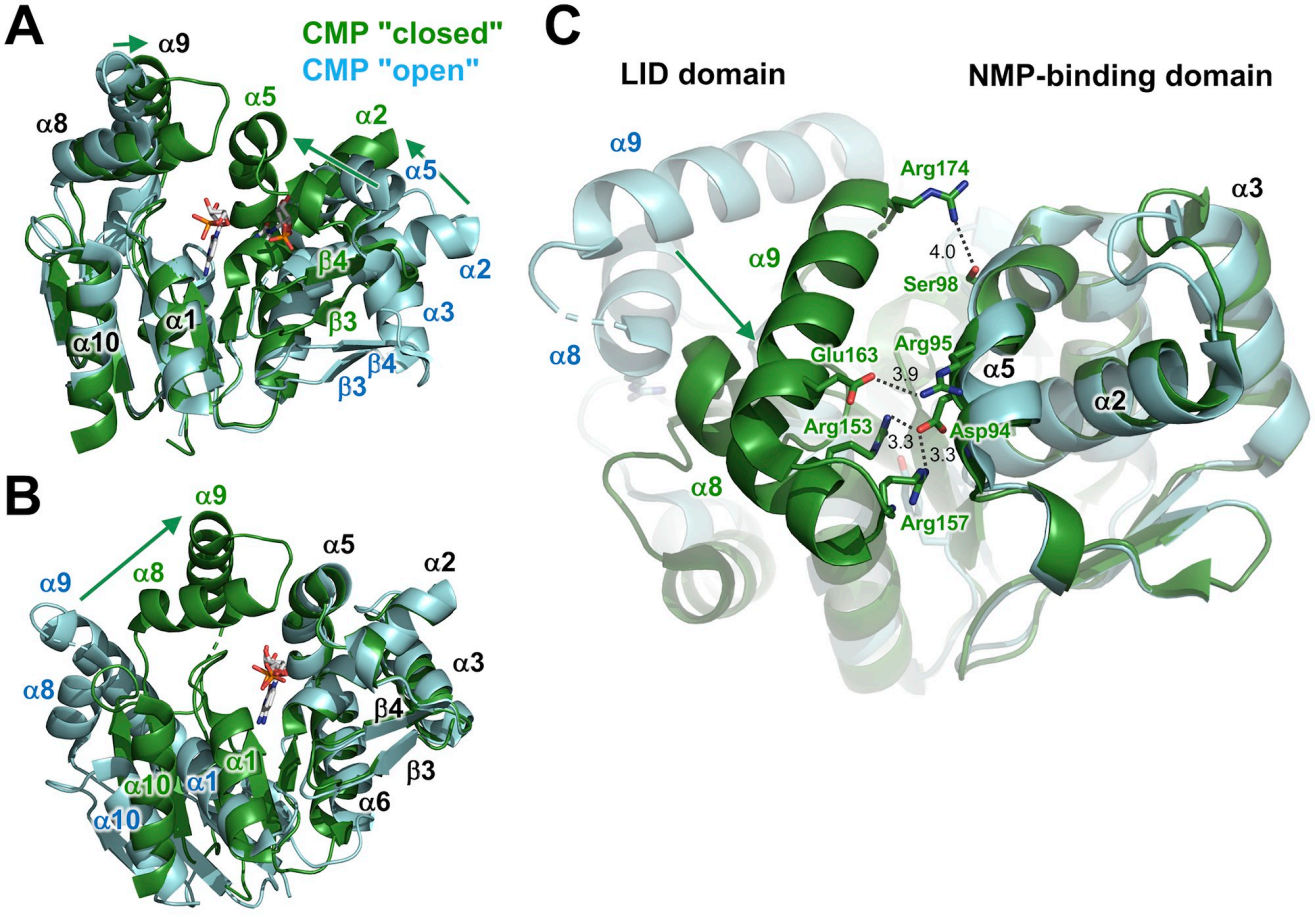
Structural comparison of the CMP "closed" complex (green) with the CMP "open" complex (cyan). (A) The structures are superimposed on the basis of C_α_ positions in the CORE domain. CMP is shown as sticks colored according to atom type. Disordered regions are shown as dotted lines. (B) The structures are superimposed on the basis of bound CMP. (C) Interaction between the NMP-binding and LID domains in the CMP "closed" complex. Compared to panel B, this figure is rotated by 80° around the horizontal axis. The side chains are shown as sticks. Numbers are the distances shown in Å.

We also compared the overall structure of the CMP "closed" complex with that of the CMP "open" complex (Fig 5A). Large shifts were also found in the position of the NMP-binding domain and LID domain relative to the CORE domain. However, the CMP molecule was bound to the NMP-binding domain similarly in both complexes, although they adopted different conformations. To make this point more clearly, these two structures were superimposed on the basis of bound CMP molecules (Fig 5B). In this superimposition, it can be shown that the closure of the cleft occurred by rotation of the CORE domain around the C-terminal end of the α10 helix as a center (pivot) point. As for the NMP-binding domain, most of the secondary structural elements (α2, α3, α5, β3, and β4) were well aligned with each other, although the C-terminal ends of the α6 helices were in a slightly different position. This clearly indicates no change in interaction of CMP to the NMP-binding domain upon the closure of the cleft. As described above, the folding of the CORE domain also shows no change (Fig 4A). Therefore, it can be said that the closure of the cleft is mainly accomplished by the movement of both domains.

Novel interactions between the NMP-binding and LID domains are thought to contribute to generation and stabilization of the closed conformation (Fig 5C). Arg95 and Ser98 of the α5 helix in the NMP-binding domain interact with Glu163 and Arg174 of the α9 helix in the LID domain, respectively. Asp 94 of the α5 helix interacts with Arg153 of the α8 helix and Arg157 in the α8-α9 loop in the LID domain. This interaction might stabilize conformation of the α8-α9 loop, part of which is disordered in the CMP "open" complex. These indicate that binding of the acceptor nucleotide to the NMP-binding domain is associated with closure of the LID domain.

**Fig 6:**
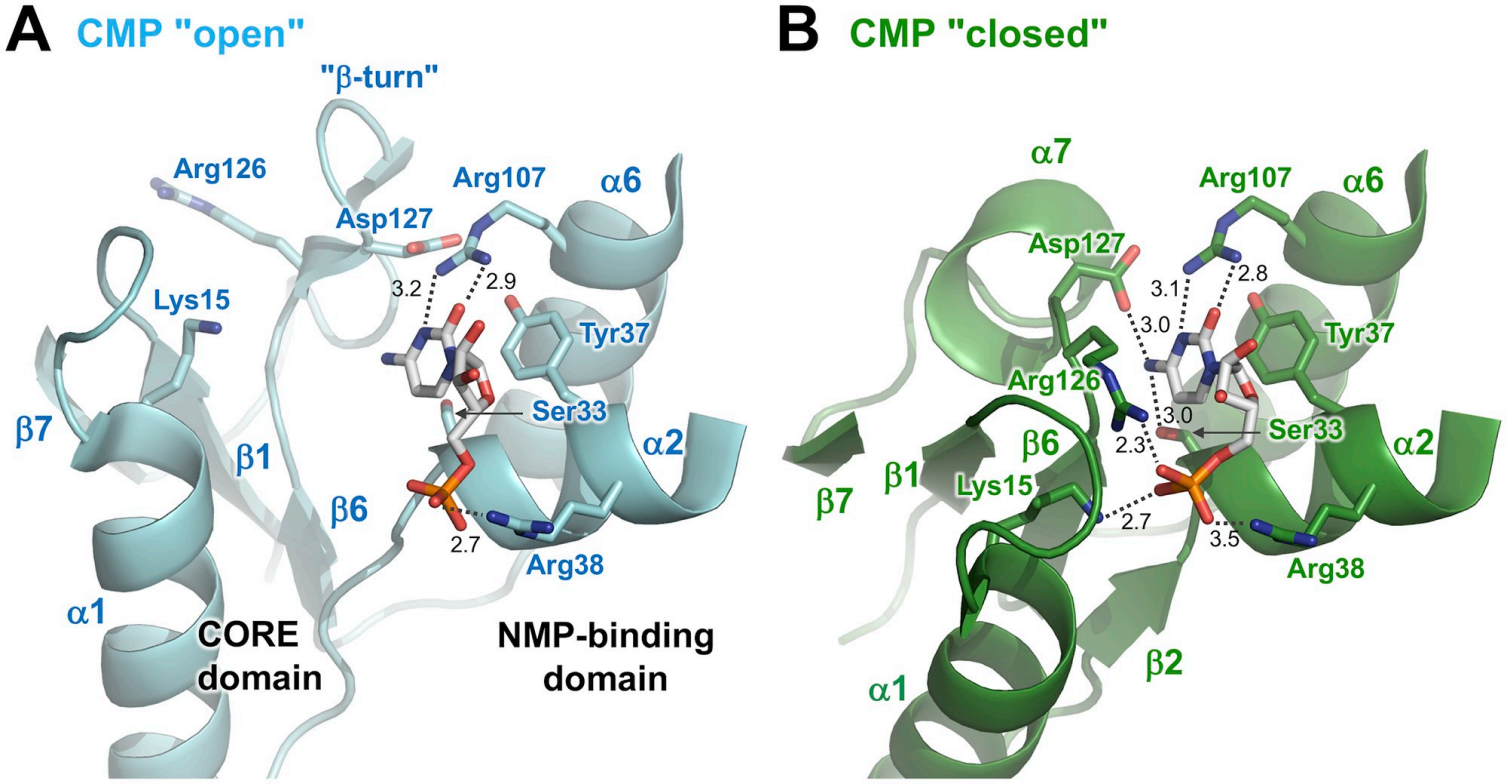
Interaction with CMP in the CMP "open" complex (A) and the CMP "closed" complex (B). CMP is shown as sticks colored according to atom type. The side chains are shown as sticks. Numbers are the distances shown in Å.

The closure of the cleft is accompanied by interaction of CMP with the CORE domain. This interaction is the main difference between these two complexes, besides the movement of domains. In the CMP "open" complex, CMP is recognized only by a few residues in the NMP-binding domain (Fig 6A). Arg107 and Arg38 interact with the cytosine and phosphate moiety of bound CMP. Tyr37 seems to bind to the cytosine ring via a stacking interaction.

In the CMP "closed" complex, Tyr37, Arg38, and Arg107 interact with CMP similarly (Fig 6B). In addition, Ser33 in the NMP-binding domain interacts with the amino group of N4 from cytosine. The side chain of Ser33 in the CMP "open" complex points in a different direction. Furthermore, three residues from the CORE domain interact with CMP in the CMP "closed" complex. Lys15 and Arg126 interact with the α-phosphate group, and Asp127 interacts with the amino group of N4 from cytosine. It can be indicated that the closure of the cleft allows interaction of CMP with these residues in the CORE domain, or these interactions cause the closure of the cleft. It should be noted that Arg126 and Asp127 are located within the β turn in the CMP "open" complex (Fig 6A) but within the α7 helix in the CMP "closed" complex (Fig 6B). The conformation of bound CMP was similar in these two structures, although the configurations of the ribose and phosphate moieties are slightly different.

### Structure of the ADP-CDP-Gd^3+^ complex

**Fig 7:**
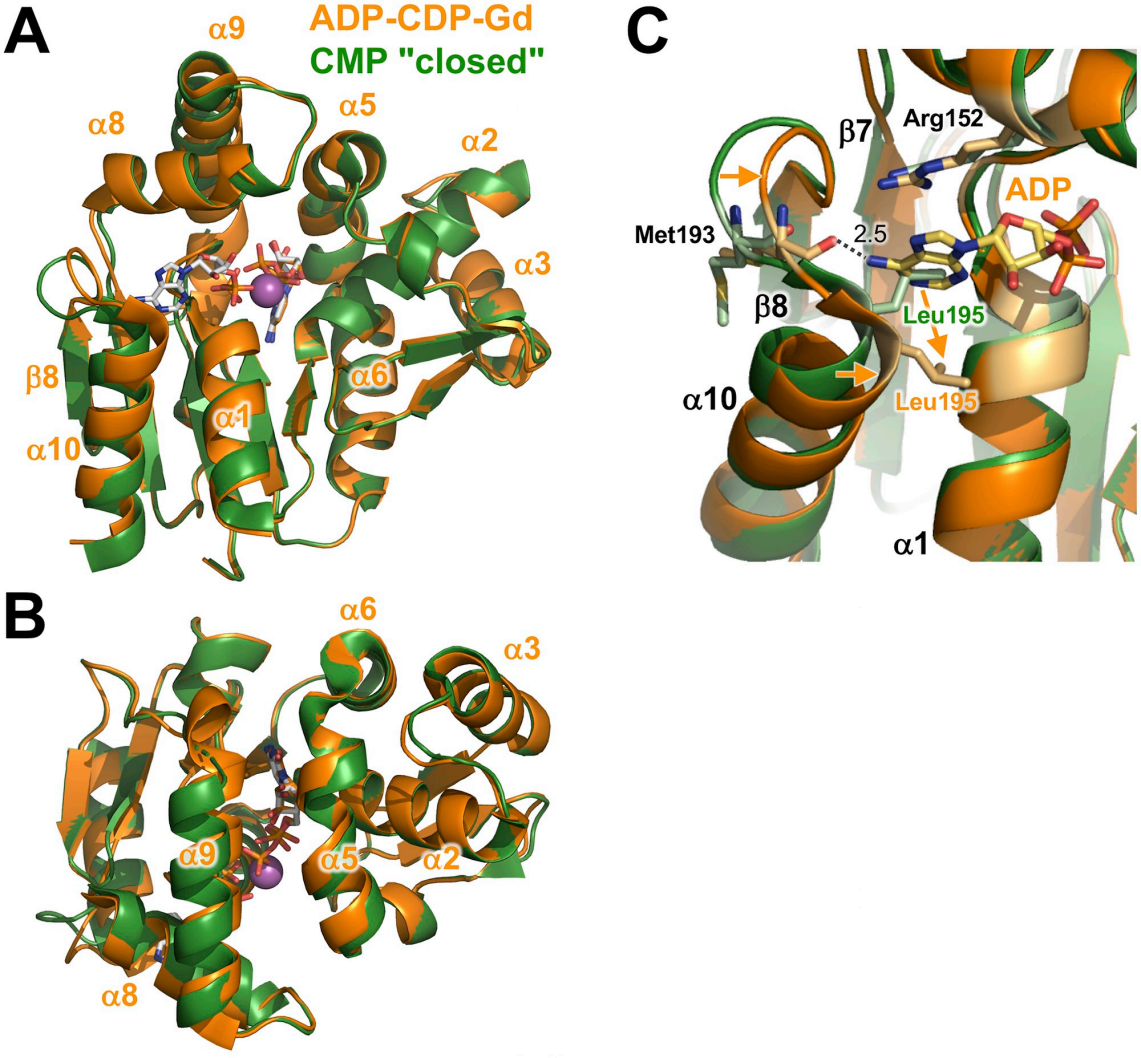
Structural comparison of the ADP-CDP-Gd^3+^ (orange) complex with the CMP "closed" complex (green). (A) The structures are superimposed on the basis of C_α_ positions in the whole proteins. ADP and CDP are shown as sticks colored according to atom type. The Gd^3+^ ion is shown as magenta sphere. Disordered regions are shown as dotted lines. (B) Compared to (A), this figure is rotated by 125° around the horizontal axis. (C) Recognition of the adenine ring of ADP.

The overall structure of the ADP-CDP-Gd^3+^ complex was almost the same as that of the CMP "closed" complex (Fig 7). The RMSD value between these two structures (206 C_α_ atoms) was 1.62 Å. Even when structural alignment was performed on the basis of C_α_ positions in the whole proteins, all three domains and bound cytidine nucleotides were well aligned. Therefore, it can be indicated that the ADP-CDP-Gd^3+^ complex is in the closed conformation as well as the CMP "closed" complex. However, a detailed comparison of both complexes revealed significant differences in the C-terminal region of the α9 helix (Fig 7B). This region is at the “bottom” of the LID domain and disordered in the CMP "closed" complex. In the ADP-CDP-Gd^3+^ complex, this region is ordered and located near the bound CDP. As described later, Asp175 and Gln178 in this region are in contact with CDP. Another difference is the linker between the β8 strand and the α10 helix (Fig 7A). In the ADP-CDP-Gd^3+^ complex, this linker moved toward bound ADP. This leads to interaction of the backbone carboxyl group of Met193 with the adenine ring (Fig 7C), as described later.

**Fig 8:**
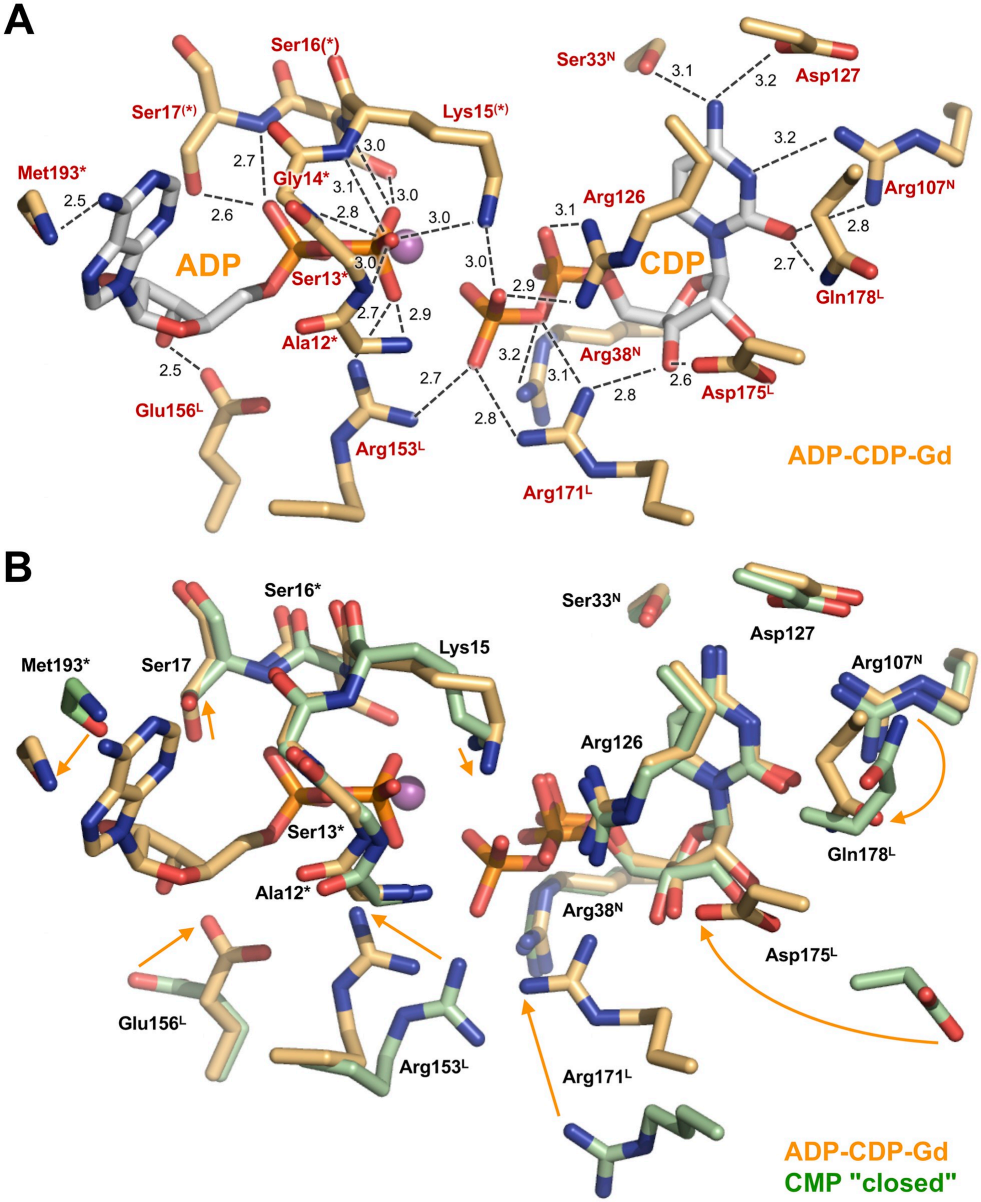
Active site in the ADP-CDP-Gd^3+^ complex. (A) Binding site of ADP and CDP. Possible interactions are shown as dashed lines. Numbers are the distances shown in Å. The Gd^3+^ ion is shown as magenta sphere. (B) Superimposition of the active site of the ADP-CDP-Gd^3+^ complex (light orange) with that of the CMP "closed" complex (light green). Note that the side chain of Leu195 is omitted from panel B because they occupy the position of the adenine ring of ADP (see Fig 7C). Superscripts L and N denote the residues from the LID and NMP-binding domains. Asterisks represent the residues whose main-chain amide groups are involved in ligand recognition. Arrows represent shifts of the side chains.

ADP is recognized by several residues from both the CORE and LID domains (Fig 8A). The phosphate moiety is surrounded by the β1-α1 loop (9-GPSASGKS-16), which is known to be a Walker A-type motif, in the CORE domain. The β-phosphate group is recognized by the main-chain amide groups of Ala12–Ser16 and the side chains of Lys15 and Ser16. In contrast, the α-phosphate is recognized only by the main-chain amide and side-chain hydroxyl groups of Ser17. The directions of the side chains of Lys15 and Ser17 are changed so as to interact with the phosphate group. In addition, the β-phosphate and hydroxyl group of ribose interact with Arg153 and Glu156 from the LID domain, respectively. The adenine base of ADP is recognized only by Met193.

The conformation of CDP is almost the same as CMP in the CMP "closed" complex except for the β-phosphate group (Fig 8B). Some of the residues recognizing CDP are common to those for CMP in the CMP "closed" complex: Ser33 and Arg107 from the NMP-binding domain and Arg126 and Asp127 from the CORE domain. Ser33 and Asp127 interact with the amino group of N4 from cytosine (Fig 8A). Arg107 interacts with both N3 and the hydroxide group of cytosine, whereas Gln178 interacts only with the hydroxide group of cytosine. Tyr37 appears to make a stacking interaction with the cytosine ring (not shown in Fig 8). Arg126 interacts with the β-phosphate in addition to the α-phosphate of CDP (Fig 8), which is similar to that with CMP (Fig 6). Furthermore, in the ADP-CDP-Gd^3+^ complex, movement of the LID domain allows the side chains of Arg171, Asp175, and Gln178 to interact with the phosphate, the hydroxyl group of ribose, and the hydroxyl group of cytosine, respectively (Fig 8). In particular, Arg171 may trigger the further closure of the LID domain upon CDP binding to the NMP-binding domain. These features reflect that CMPK could have a strong specificity to bind CMP/CDP compared to ATP/ADP. Lys15 and Arg153 also interact with the β-phosphate group of CDP, in addition to the β-phosphate group of ADP. Especially, recognition by these two resides might be important for catalytic reaction because the terminal phosphate of CDP is transferred from ATP.

Arg153, Glu156, Arg171, Arg175, and Gln178 from the LID domain show large shifts of their side chains when the ADP-CDP-Mg and CMP "closed" complexes are compared (Fig 8B). Arg153 and Glu156 are in the linker region between the α8 and α9 helices, whereas the other three are in the α9 helix. This means that conformational change of the LID domain enabled these residues to interact with the nucleotides; in other words, binding of both ADP and CDP led to a more closed conformation.

In addition, as described above, conformational change of the linker region between the β8 and α10 (Fig 7C) led to interaction of the Met193 main-chain carbonyl group with the adenine ring (Fig 8B). This might be caused by a slight shift of the N-terminal region of the α10 (Fig 7C). This shift seems to be linked to movement of the side chain of Leu195. The side chain of Leu195 in the CMP "closed" complex occupied the position of the adenine ring of ADP in the ADP-CDP-Gd^3+^ complex (Fig 7C). Upon binding of ADP, the position of this side chain might be changed, so as to avoid steric hindrance. As a result, the side chain of Leu195 seems to form a hydrophobic interaction with the adenine ring in the ADP-CDP-Gd^3+^ complex. It should be mentioned that ADP in the ADP-CDP-Gd^3+^ complex was in the *syn* conformation. Interaction of Met193 with the amino group of the adenine might stabilize ADP in the *syn* conformation.

**Fig 9:**
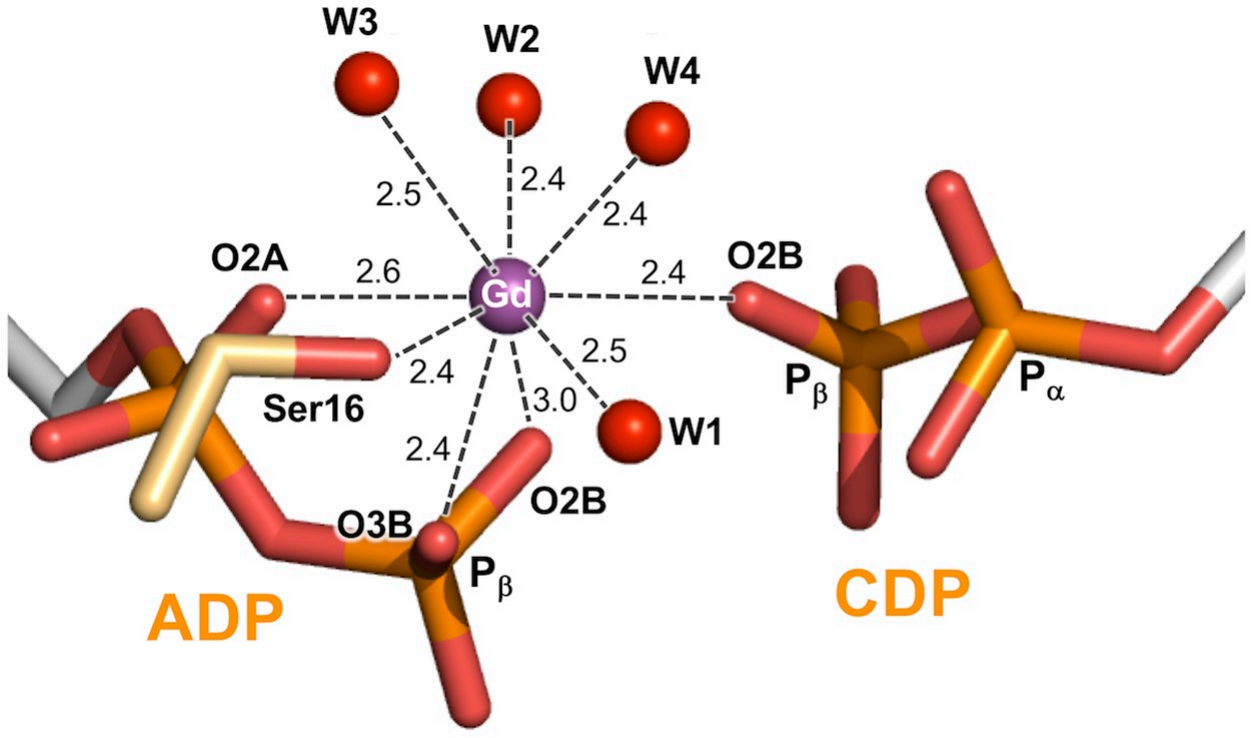
Coordination of the Gd^3+^ ion in the ADP-CDP-Gd^3+^ complex. Oxygen atoms of water molecules and Gd^3+^ ion are shown as red and magenta spheres, respectively. Possible interactions are shown as dashed lines. Numbers are the distances shown in Å.

The Gd^3+^ ion is in contact with the β-phosphate groups from both ADP and CDP (Fig 9). It is coordinated with the O2A oxygen atom of the α-phosphate and two oxygen atoms of the β-phosphate from ADP, the O2B atom of the β-phosphate from CDP, the carboxylate of the side chain of Ser16, and four water molecules. It is known that the Gd^3+^ ion can form complexes having high coordination numbers up to nine [39]. Two of the four water molecules interact with both ADP and CDP. Furthermore, one of the two water molecules (W1) interacts with the carboxylate of Glu124.

In this crystal structure, one terminal oxygen (O2B) of ADP directly points to the phosphorus atom of the β-phosphate of CDP at a distance of 3.4 Å and in line with the P_β_–O_α,β_ bond (Fig 9). This geometry strongly suggests a direct nucleophilic attack of O2B on the P_β_ atom of ADP, following an associative mechanism for phosphoryl transfer. The distribution of positive charges in the active site further supports this model (Fig 8A). The side chains of Arg153 and Arg171 bind to the O3B oxygen of transferable phosphate. Lys15 in the P-loop bridges the ADP and CDP via interaction with the respective O1B oxygens. These basic residues seem to be well positioned to neutralize the negative charge developed on the putative trigonal bipyramid transition state.

### Structure of the CDP complex

**Fig 10:**
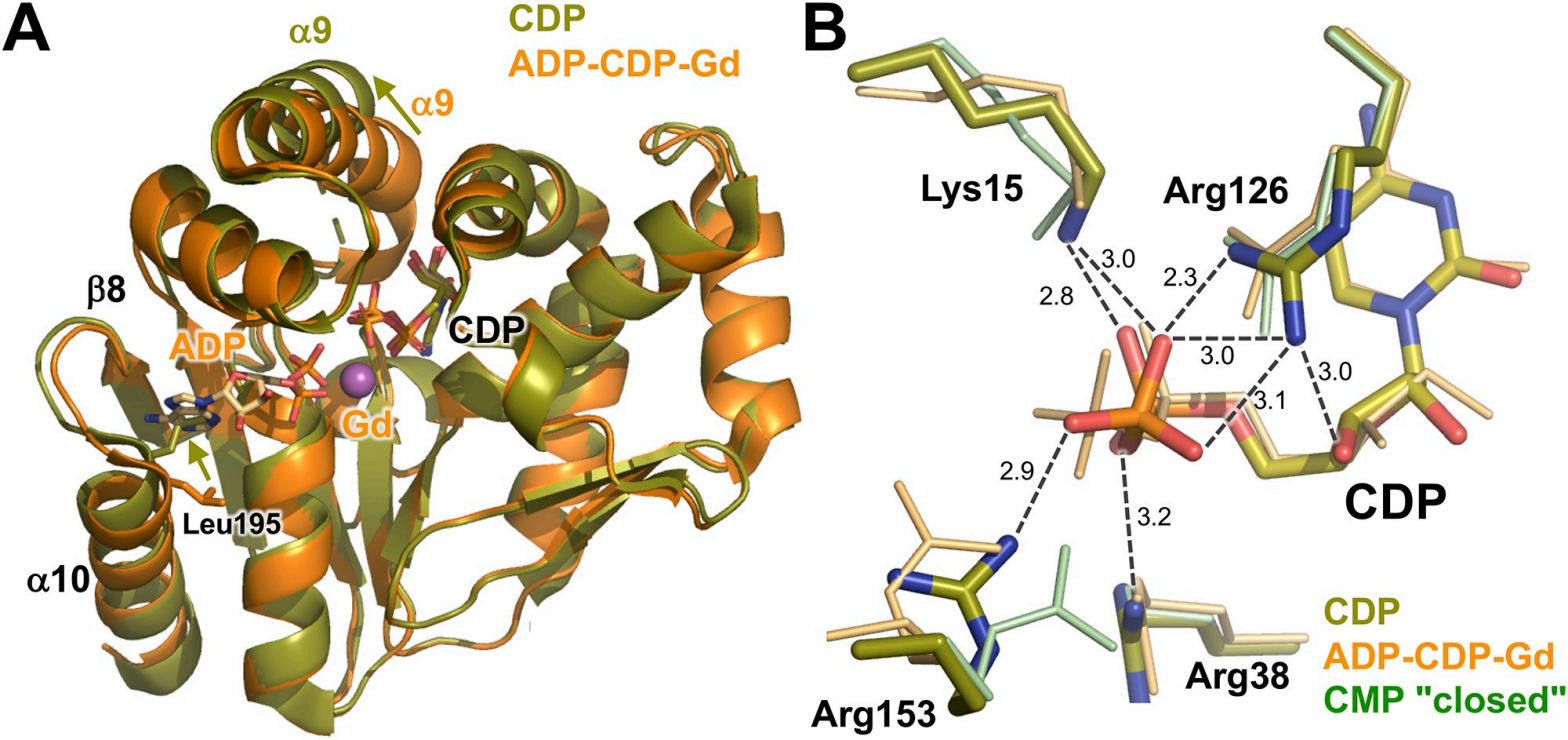
Structure of the CDP complex. (A) The structures of the CDP complex (olive) and ADP-CDP-Gd^3+^ complex (orange) are superimposed on the basis of C_α_ positions in the whole proteins. ADP and CDP are shown as sticks. The side chains of Leu195 are shown as sticks. (B) Recognition of CDP. CDP and selected residues are colored as follows: olive, CDP complex; light orange, ADP-CDP-Gd^3+^ complex; and light green, CMP "closed" complex. The CDP and residues from the CDP complex are shown as thick sticks. Numbers are the distances shown in Å.

The structure of CDP-complex was almost the same as that of the CMP "closed" complex: both were in the closed conformation. The RMSD value between these two structures (202 C_α_ atoms) was 0.81 Å. Therefore, the differences between the CDP "open" complex and the ADP-CDP-Gd^3+^ complex (the RMSD value of 1.48 Å; 199 C_α_ atoms) were similar to those between the CMP "closed" complex and the ADP-CDP-Gd^3+^ complex (the RMSD value of 1.62 Å; 206 C_α_ atoms). The major differences are observed in the C-terminal region of the α9 helix and the linker between the β8 strand and the α10 helix (Fig 10A). Accompanied with the motion in the latter region, the side chain of Leu195 was moved back to the position this residue occupied in the CMP "closed" complex. However, the conformation and recognition of the terminal phosphate group of CDP were changed (Fig 10B). The difference in the angle of the P_β_–O_α,β_ bond was approximately 60 degrees: the β-phosphate in the CDP complex did not direct toward the ADP-binding site. This conformation is considered to be maintained by several interactions specific to the CDP complex. Lys15 interacts with the α-phosphate oxygen, in addition to the β-phosphate oxygen. Arg126 interacts with the hydroxy group of the ribose, in addition to the α- and β-phosphate oxygens. The side chain of Arg153 in the LID domain showed a significant but less shift than in the CMP "closed" complex and still interacts with the β-phosphate oxygen. The side chain of Arg171 in the LID domain was moved away from CDP, which led to loss of interaction with the phosphate group.

## Discussion

In this study, we determined structures of the ligand-free, CMP-bound, CDP-bound, and ADP-CDP-bound forms of ttCMPK. In particular, we determined for the first time the crystal structure of CMPK with adenine nucleotide bound in the phosphate donor site. This study enabled us to compare these structures and to elucidate conformational changes upon binding of ligands in the reaction pathway (Fig 2).

### Ligand-free forms of CMPKs

**Fig 11:**
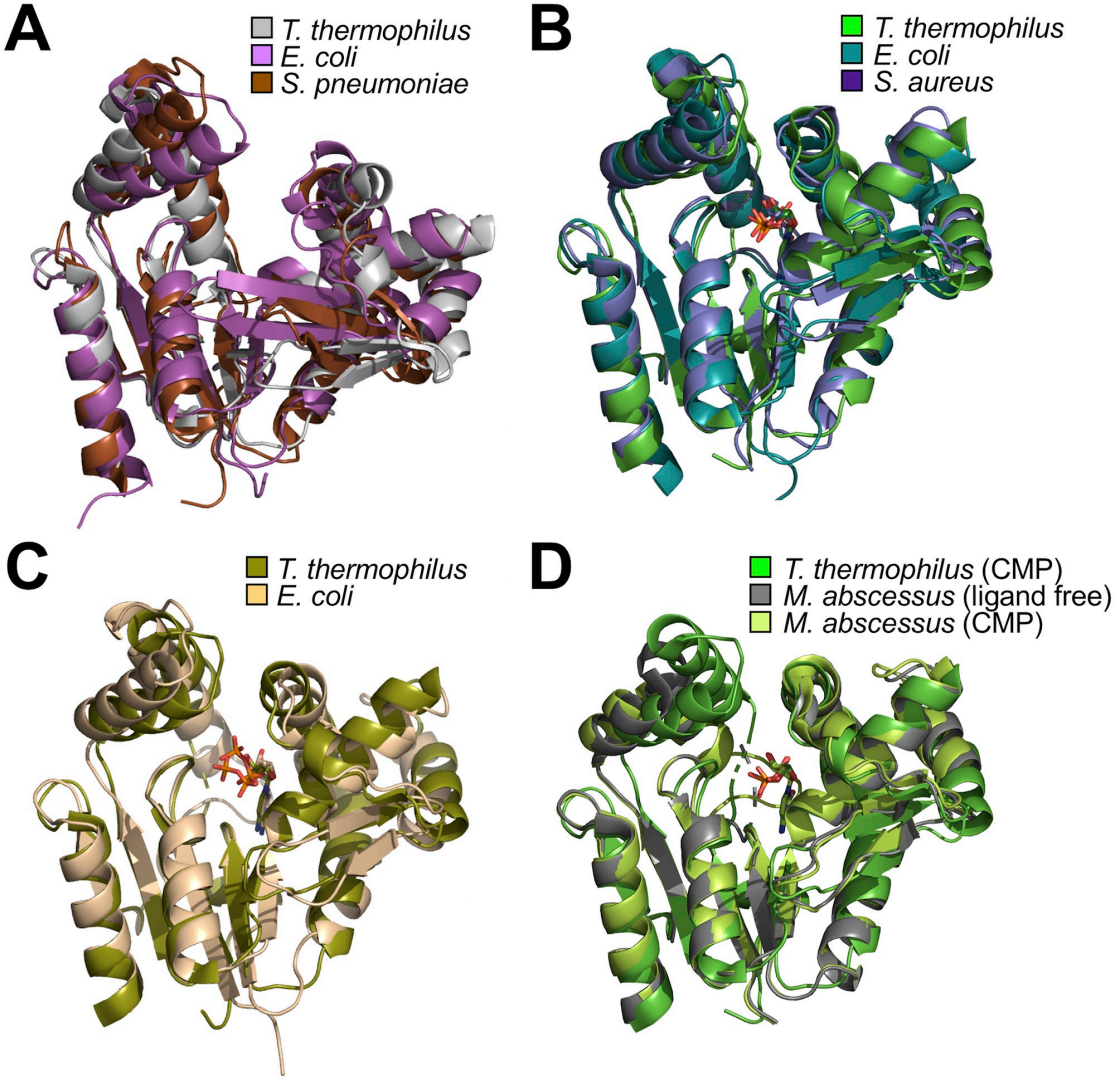
Comparison of CMPK structures. (A) Ligand-free forms of CMPK from *T*. *thermophilus* (gray; PDB code 3W90), *E*. *coli* (magenta; 1CKE), and *S*. *pneumoniae* (brown; 1Q3T). (B) CMP-bound forms of CMPK from *T*. *thermophilus* (green; 3AKE), *E*. *coli* (teal; 1KDO), and *S*. *aureus* (light purple; 2H92). (C) CDP-bound forms of CMPK from *T*. *thermophilus* (olive; 3AKD) and *E*. *coli* (light brown; 2CMK). (D) CMP-bound forms of CMPK from *T*. *thermophilus* (green; 3AKE) and *M*. *abscessus* (light green; 4DIE), and ligand-free form of CMPK from *M*. *abscessus* (dark gray; 3R8C). These structures were structurally aligned based on superimposing the backbone structure of their CORE domains. For ttCMPK, the CMP "closed" complex was employed as CMP-bound form.

The structures of CMPK in an ligand-free form have been determined for CMPKs from *E*. *coli*, *S*. *pneumoniae*, *Y*. *pseudotuberculosis*, *M*. *smegmatis*, and *M*. *abscessus* [19–21,24]. Fig 11A shows structural comparison of the ligand-free forms from ttCMPK, *E*. *coli*, and *S*. *pneumoniae* CMPKs. The structure of *Y*. *pseudotuberculosis* CMPK is not shown in Fig 11A because it is very similar to *E*. *coli* CMPK. When the CORE domains were superimposed, the NMP-binding and LID domains were not well aligned, although their secondary structure elements are almost the same. For *E*. *coli* CMPK, the LID domain and the two β-strands in the NMP-binding domain are in a more closed conformation compared to the other two CMPKs. NMR relaxation studies of *S*. *pneumoniae* CMPK (ligand-free form) have showed that the loops in the LID domain are highly mobile [24]. Also in our study, the loop between the α8 and α9 helices in the LID domain was disordered in the ligand-free form and had a higher B-factor than the other regions (Fig 3). These support the notion that the loop in the LID domain is mobile. Therefore, it is suggested that the ligand-free form can be in a variety of open conformations, and arrangement of the domains are not rigid within the range of open conformation.

### CMP/CDP-bound forms of CMPKs

In contrast, the structures of the CMP-bound forms of three CMPKs [20,22,23] were well aligned, as shown in Fig 11B. The structural variations in the LID domains were relatively small compared to those in ligand-free forms. Also, the CDP-bound forms showed similar structures for ttCMPK and *E*. *coli* CMPK [19] (Fig 11C). The positions of the bound CMP and CDP were also the same in the respective complexes, although the direction of the phosphate groups of CDP were significantly different. Only NMP domain moves upon binding of CDP to *E*. *coli* CMPK [19]. However, in the case of ttCMPK, upon binding of the phosphoryl acceptor, CMP or CDP, the LID domain moved as well as the NMP domain (Figs 4 and 10). This apparent discrepancy may be due to fluctuations of the LID domain described above. Actually, for *E*. *coli* CMPK, the α helix in the LID domain, corresponding to the α8 helix in ttCMPK, is in a more closed conformation in the ligand-free form than those in the CMP- and CDP-bound forms (Fig 11B and 11C), which is opposite in direction to ttCMPK (Fig 1B).

**Fig 12:**
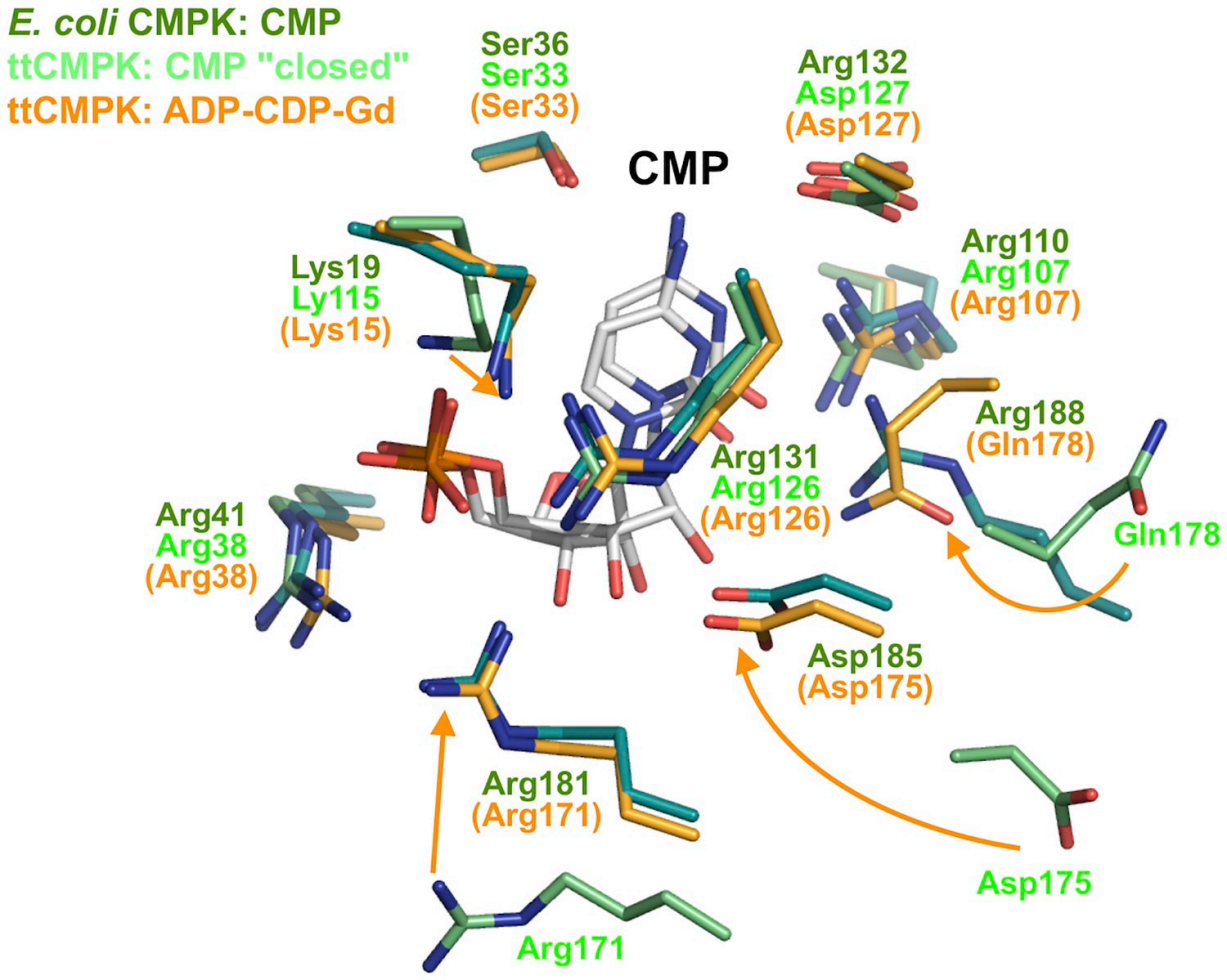
Comparison of CMP-binding sites between ttCMPK and *E*. *coli* CMPK. The side chains are colored as follows: the CMP complex of *E*. *coli* CMPK (in dark green), the CMP "closed" complex of ttCMPK (in green), and the ADP-CDP-Gd^3+^ complex of ttCMPK (in orange). The names of residues in the ADP-CDP-Gd^3+^ complex are represented in parentheses. Arrows represent shifts of the side chains in the CMP "closed" complex to the ADP-CDP-Gd^3+^ complex of ttCMPK.

Most of the residues interacting with bound CMP are conserved among ttCMPK, *E*. *coli*, and *S*. *pneumoniae* CMPKs. Fig 12 shows interaction with CMP in ttCMPK and *E*. *coli* CMPK. However, the positions of Arg181, Asp185, and Arg188 of *E*. *coli* CMPK are largely different from those of the corresponding residues (Arg171, Asp173, and Gln178) of ttCMPK in the CMP "closed" complex but are similar to those of these residues in the ADP-CDP-Gd^3+^ complex. The side chains of these three residues were shifted and accompanied by binding of ADP. As for the CMP-binding site, which is formed by the three domains, the conformation of the *E*. *coli* CMP-bound form seems to correspond to that of the ttCMPK ADP-CDP-bound form. Therefore, this difference between ttCMPK and *E*. *coli* CMPK may be due to fluctuation of the side chains within the LID domain. In other words, the observed conformational changes in ttCMPK structures might represent fundamental processes of ligand binding.

An intriguing observation in this study is the CMP-bound forms were in the open and closed conformations (Fig 5). This is the first reported crystal structure of CMPK in the open conformation with a ligand bound. These structures may be biologically relevant conformations and true transition intermediates that have been stabilized by crystal contacts. In the case of AMPK, which belongs to the same protein family as CMPK, experiments and computational studies have shown the open−closed conformational change can occur even in the absence of ligands [6,9–13]. For the ligand-bound AMPK, the free energy difference between the open and closed states is suggested to be ~5 kcal/mol by some simulation studies [8,40]. Such relatively low energy barriers would allow the enzyme to sample both the open and closed states. Small-angle X-ray scattering experiments for AMPK showed the open conformation is dominant even at high AMP concentrations [14]. In general, however, it is difficult to say how such structural polymorphisms relate to ensembles of protein structures [41]. Alternatively, the observed structural variation may arise due to differences in crystal packing instead of representing natural protein dynamics. In connection with the open–closed transition, it is noteworthy that the ligand-free and CMP-bound forms of *M*. *abscessus* CMPK have the same conformation (Fig 11D) [20]. The ligand-free form of *M*. *smegmatis* CMPK (PDB code 3R20) is almost the same structure as *M*. *abscessus* CMPK. Interestingly, these conformations of *Mycobacterium* CMPKs are similar to that of ttCMPK in the CMP "closed" complex and the CDP complex (Fig 11D). Therefore, it can be considered that the ligand-free forms of *Mycobacterium* CMPKs are in a closed conformation. This implies that CMPK can take a closed conformation even in the absence of ligands. A closed conformation in an ligand-free enzyme is thought to be a prerequisite for the conformational selection model, as discussed later [16]. In the case of AMPK, NMR experiments have indicated that closed conformations exist in the absence of ligands [5,6].

It should be noted that the crystallization solutions for the CMP "closed" and CDP complexes contained AMP-PCP and ATP (and probably ADP as a reaction product), respectively. There is the possibility that binding of AMP-PCP or ADP stabilized the closed conformation. However, traceable electron density of these nucleotides was not achieved with the X-ray data sets of these crystals. In addition, significant movement of the side chains was not observed in the ATP-binding site. Based on these results, we consider that the above possibility is not completely excluded but unlikely. Therefore, we argue that CMP or CDP binding alone can lead to a closed conformation.

### ADP-bound form of CMPK

We further determined for the first time the crystal structure of CMPK with adenine nucleotide–bound in the phosphate donor site. The overall conformation of the ADP-CDP-bound form was very similar to those of the CMP "closed" and CDP complexes (Figs 7 and 10). However, significant difference in the overall fold was observed in the C-terminal region of the α9 helix in the LID domain. This region was ordered in the electron-density map and moved toward CDP in the ADP-CDP complex compared to in the CMP "closed" complex (Fig 7B). Candidate residues that play key roles in this movement may be Arg171, Asp175, and Gln178, which are located in the C-terminal region of the α9 helix in the LID domain. The side chains of these three residues were shifted to interact with CMP accompanied by binding of ADP (Fig 8). However, it should be noted that these residues have no interaction with ADP. It is uncertain how the binding of ADP triggered the shift of these residues. Arg153 and Glu156 in the α8 helix in the LID domain may be involved in this process. These residues were moved toward and interacted with ADP (Fig 8), although the α8 helix showed little shift upon ADP binding (Fig 7).

**Fig 13:**
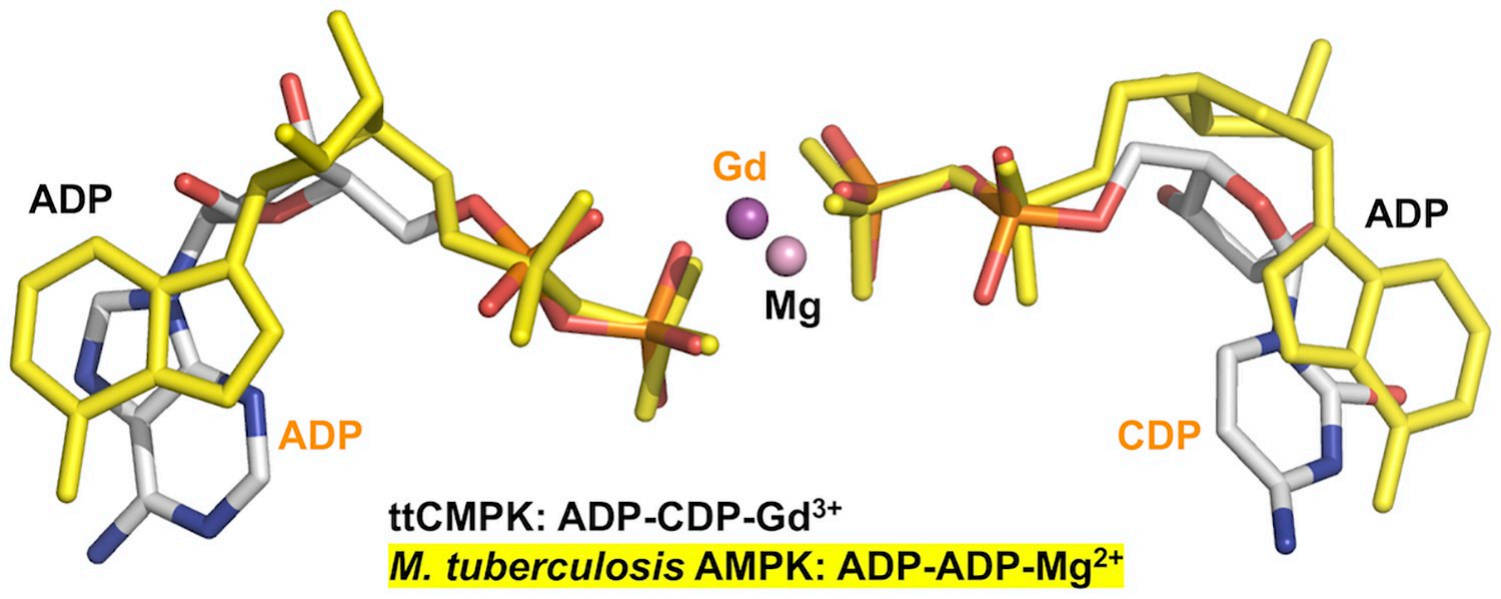
Bound nucleotides in the ternary complexes of ttCMPK (white) and *Mycobacterium tuberculosis* AMPK (yellow). The nucleotides bound to ttCMPK are shown in the CPK model. Gd^3+^ and Mg^2+^ ions are in ttCMPK and *Mycobacterium* AMPK, respectively. The PDB codes for these structures are 3AKC (this study) and 2CDN [43].

Another small but significant change upon ADP binding was observed around the N-terminal region of the α10 helix (Fig 7C). This region might be moved to avoid steric clash of the side chain of Leu195 with the adenine ring of ADP. This might also lead to interaction of the Met193 main-chain carbonyl group with the exocyclic amino group of the adenine moiety (Fig 7C). In connection with recognition of ADP, it should be noted that the conformation of ADP in ttCMPK is different from that in AMPK. In the ADP-CDP-Gd^3+^ complex of ttCMPK, ADP is in the *syn*-form with respect to the N-glycosidic bond between adenine and ribose moieties (Figs 8 and 13). In contrast, in the ADP complex of AMPK, ADP in the phosphoryl donor site is in the *anti*-form (Fig 13). The *syn*-form of ADP may be stabilized by Leu195 and Met193 in ttCMPK (Fig 7C). These structural features may explain the relatively high specificity of CMPKs toward the nucleotide bound to the ATP site [42]. In the case of *Mycobacterium* AMPK, the exocyclic amino group of ADP in the ATP site forms a single hydrogen bond with the carbonyl oxygen of Gly. This can explain the relatively poor specificity of AMPKs toward the nucleotide bound to the ATP site [43].

The geometry of the bound nucleotides suggests a direct nucleophilic attack to the phosphate group in an in-line displacement mechanism (Figs 9 and 13). The distance between the two P_β_ atoms is 4.5 Å, which is similar to that in AMPK [44]. Some interactions observed in the ttCMPK crystal structure are thought to be involved in the correct positioning and stabilization of the negatively charged phosphoryl group (Fig 8A). The terminal phosphate groups of ADP and CDP in ttCMPK have similar geometry to those of two ADP in AMPK (Fig 13), suggesting a common catalytic mechanism for the NMP kinase family [44]. A structure of ttCMPK transition-state analogue complex should help to reveal the catalytic mechanism.

### Coupling of conformational changes with ligand binding

Comparison of the determined structures revealed both global and local changes in ttCMPK structure along the reaction pathway. Fig 2 shows a schematic representation of the observed or hypothetical conformational changes upon substrate binding. Global changes in protein structure usually involve hinge motions or rotation of multiple domains toward one another, resulting in the closure of the active-site cleft [45]. Two extreme mechanisms have been proposed for such changes: induced-fit mechanism and conformational selection (population shift) mechanism [16–18]. In the former, binding of substrates induces a change in conformation, and optimal binding is achieved by specific structural change. In the latter, binding of substrates shifts the equilibrium distribution of structures and traps the protein in a particular conformational state among the already present unbound ensemble. Many studies have been performed to determine whether ligand binding to AMPK occurs with induced-fit or conformational selection pathways [6,8,10–15]. However, the complexity of this problem has been left open. In ttCMPK, global open−closed conformational changes involving the three domains were observed upon binding of CMP or CDP. On the other hand, the side chains of several residues were moved toward the nucleotides without global conformational changes upon simultaneous binding of ADP and CDP (Fig 8). It is uncertain at present whether these global and local changes occur via induced-fit mechanism or conformational selection mechanism. In this regard, it could be said that the observation that the CMP-bound form took an open conformation (Fig 5) is consistent with conformational selection mechanism. The closed conformation of *Mycobacterium* CMPK in the absence of ligand (Fig 11D) also supports conformational selection mechanism. However, the shift of the Leu195 side chain seems to be induced by binding of ADP (Fig 7C). The reaction catalyzed by CMPK may proceed according to a hybrid model involving both mechanisms, which has been proposed for AMPK [46].

## Supporting information

S1 Data (PDF)

S2 Data (PDF)

S3 Data (PDF)

S4 Data (PDF)

S5 Data (PDF)
