# Supplementary material for: The crystal structures of Thermus thermophilus CMP kinase complexed with a phosphoryl group acceptor and donor
Source: PLoS One. 2020 May 29;15(5):e0233689. doi: 10.1371/journal.pone.0233689 (PMC7259600; doi:10.1371/journal.pone.0233689)
Supplement: S5 Data — (PDF) [file pone.0233689.s005.pdf]

Full wwPDB X-ray Structure Validation Report ⓘ

Mar 8, 2018 – 11:05 pm GMT

PDB ID	:	3W90
Title	:	Crystal structure of CMP kinase from Thermus thermophilus HB8
Authors	:	Nakagawa, N.; Mega, R.; Masui, R.; Kuramitsu, S.; RIKEN Structural Genomics/Proteomics Initiative (RSGI)
Deposited on	:	2013-03-22
Resolution	:	1.65 Å(reported)

This is a Full wwPDB X-ray Structure Validation Report for a publicly released PDB entry.

We welcome your comments at *validation@mail.wwpdb.org*
A user guide is available at
https://www.wwpdb.org/validation/2017/XrayValidationReportHelp
with specific help available everywhere you see the ⓘ symbol.

The following versions of software and data (see references ⓘ) were used in the production of this report:

MolProbity	:	4.02b-467
Xtriage (Phenix)	:	1.13
EDS	:	trunk30967
Percentile statistics	:	20171227.v01 (using entries in the PDB archive December 27th 2017)
Refmac	:	5.8.0158
CCP4	:	7.0 (Gargrove)
Ideal geometry (proteins)	:	Engh & Huber (2001)
Ideal geometry (DNA, RNA)	:	Parkinson et al. (1996)
Validation Pipeline (wwPDB-VP)	:	trunk30967

1 Overall quality at a glance ⓘ

The following experimental techniques were used to determine the structure:
X-RAY DIFFRACTION

The reported resolution of this entry is 1.65 Å.

Percentile scores (ranging between 0-100) for global validation metrics of the entry are shown in the following graphic. The table shows the number of entries on which the scores are based.

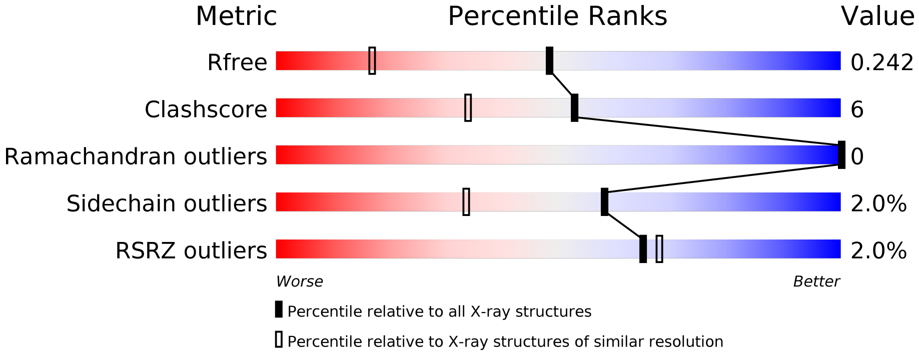

Metric	Whole archive (#Entries)	Similar resolution (#Entries, resolution range(Å))
R_{free}	111664	1521 (1.66-1.66)
Clashscore	122126	1616 (1.66-1.66)
Ramachandran outliers	120053	1584 (1.66-1.66)
Sidechain outliers	120020	1584 (1.66-1.66)
RSRZ outliers	108989	1487 (1.66-1.66)

The table below summarises the geometric issues observed across the polymeric chains and their fit to the electron density. The red, orange, yellow and green segments on the lower bar indicate the fraction of residues that contain outliers for >=3, 2, 1 and 0 types of geometric quality criteria. A grey segment represents the fraction of residues that are not modelled. The numeric value for each fraction is indicated below the corresponding segment, with a dot representing fractions <=5% The upper red bar (where present) indicates the fraction of residues that have poor fit to the electron density. The numeric value is given above the bar.

Mol	Chain	Length	Quality of chain
1	A	208	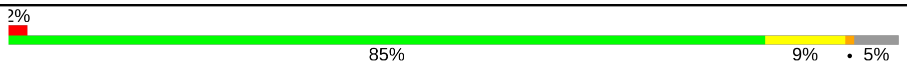

2 Entry composition (i)

There are 2 unique types of molecules in this entry. The entry contains 1676 atoms, of which 0 are hydrogens and 0 are deuteriums.

In the tables below, the ZeroOcc column contains the number of atoms modelled with zero occupancy, the AltConf column contains the number of residues with at least one atom in alternate conformation and the Trace column contains the number of residues modelled with at most 2 atoms.

- Molecule 1 is a protein called Cytidylate kinase.

Mol	Chain	Residues	Atoms					ZeroOcc	AltConf	Trace
			Total	C	N	O	S			
1	A	198	1506	951	279	274	2	0	0	0

- Molecule 2 is water.

Mol	Chain	Residues	Atoms		ZeroOcc	AltConf
			Total	O		
2	A	170	170	170	0	0

3 Residue-property plots (i)

These plots are drawn for all protein, RNA and DNA chains in the entry. The first graphic for a chain summarises the proportions of the various outlier classes displayed in the second graphic. The second graphic shows the sequence view annotated by issues in geometryand electron density. Residues are color-coded according to the number of geometric quality criteria for which they contain at least one outlier: green = 0, yellow = 1, orange = 2 and red = 3 or more. A red dot above a residue indicates a poor fit to the electron density (RSRZ > 2). Stretches of 2 or more consecutive residues without any outlier are shown as a green connector. Residues present in the sample, but not in the model, are shown in grey.

• Molecule 1: Cytidylate kinase

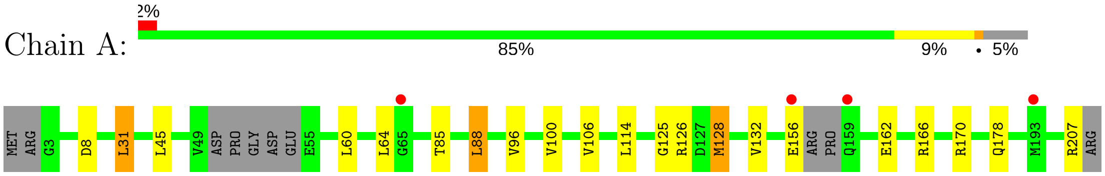

4 Data and refinement statistics

Property	Value	Source
Space group	P 21 21 21	Depositor
Cell constants<br>a, b, c, α, β, γ	35.73Å 48.38Å 110.03Å<br>90.00° 90.00° 90.00°	Depositor
Resolution (Å)	44.29 – 1.65<br>44.29 – 1.60	Depositor<br>EDS
% Data completeness<br>(in resolution range)	98.7 (44.29-1.65)<br>98.3 (44.29-1.60)	Depositor<br>EDS
R_{merge}	0.06	Depositor
R_{sym}	(Not available)	Depositor
$< I/\sigma(I) >$ [1]	6.18 (at 1.60Å)	Xtriage
Refinement program	CNS 1.3	Depositor
R, R_{free}	0.214 , 0.245<br>0.211 , 0.242	Depositor<br>DCC
R_{free} test set	2557 reflections (9.85%)	wwPDB-VP
Wilson B-factor (Å^2)	17.9	Xtriage
Anisotropy	0.150	Xtriage
Bulk solvent k_{sol}(e/Å^3), B_{sol}(Å^2)	0.35 , 50.6	EDS
L-test for twinning[2]	$< \|L\| >$ = 0.49, $< L^2 >$ = 0.33	Xtriage
Estimated twinning fraction	No twinning to report.	Xtriage
F_o,F_c correlation	0.95	EDS
Total number of atoms	1676	wwPDB-VP
Average B, all atoms (Å^2)	21.0	wwPDB-VP

Xtriage's analysis on translational NCS is as follows: *The largest off-origin peak in the Patterson function is 9.30% of the height of the origin peak. No significant pseudotranslation is detected.*

[1]Intensities estimated from amplitudes.

[2]Theoretical values of $< |L| >$, $< L^2 >$ for acentric reflections are 0.5, 0.333 respectively for untwinned datasets, and 0.375, 0.2 for perfectly twinned datasets.

5 Model quality (i)

5.1 Standard geometry (i)

The Z score for a bond length (or angle) is the number of standard deviations the observed value is removed from the expected value. A bond length (or angle) with $|Z| > 5$ is considered an outlier worth inspection. RMSZ is the root-mean-square of all Z scores of the bond lengths (or angles).

Mol	Chain	Bond lengths		Bond angles	
		RMSZ	#$\|Z\|$ >5	RMSZ	#$\|Z\|$ >5
1	A	0.87	1/1532 (0.1%)	0.95	0/2080

All (1) bond length outliers are listed below:

Mol	Chain	Res	Type	Atoms	Z	Observed(Å)	Ideal(Å)
1	A	128	MET	SD-CE	-5.31	1.48	1.77

There are no bond angle outliers.

There are no chirality outliers.

There are no planarity outliers.

5.2 Too-close contacts (i)

In the following table, the Non-H and H(model) columns list the number of non-hydrogen atoms and hydrogen atoms in the chain respectively. The H(added) column lists the number of hydrogen atoms added and optimized by MolProbity. The Clashes column lists the number of clashes within the asymmetric unit, whereas Symm-Clashes lists symmetry related clashes.

Mol	Chain	Non-H	H(model)	H(added)	Clashes	Symm-Clashes
1	A	1506	0	1532	18	0
2	A	170	0	0	4	0
All	All	1676	0	1532	18	0

The all-atom clashscore is defined as the number of clashes found per 1000 atoms (including hydrogen atoms). The all-atom clashscore for this structure is 6.

All (18) close contacts within the same asymmetric unit are listed below, sorted by their clash magnitude.

Atom-1	Atom-2	Interatomic distance (Å)	Clash overlap (Å)
1:A:128:MET:HE1	1:A:132:VAL:HG21	1.71	0.71

Continued on next page...

Continued from previous page...

Atom-1	Atom-2	Interatomic distance (Å)	Clash overlap (Å)
1:A:31:LEU:HD21	1:A:114:LEU:HD22	1.78	0.65
1:A:100:VAL:HG12	1:A:106:VAL:HG11	1.78	0.65
1:A:128:MET:CE	1:A:132:VAL:HG21	2.29	0.61
1:A:45:LEU:HD21	1:A:96:VAL:HG21	1.82	0.61
1:A:31:LEU:C	1:A:31:LEU:HD23	2.22	0.59
1:A:207:ARG:NH2	2:A:313:HOH:O	2.37	0.58
1:A:128:MET:HE1	1:A:132:VAL:CG2	2.37	0.55
1:A:128:MET:HE2	2:A:412:HOH:O	2.06	0.54
1:A:85:THR:HA	1:A:88:LEU:HD22	1.90	0.54
1:A:170:ARG:HD3	2:A:421:HOH:O	2.08	0.52
1:A:60:LEU:HD11	1:A:64:LEU:HD11	1.95	0.49
1:A:8:ASP:HA	1:A:125:GLY:O	2.12	0.49
1:A:128:MET:CE	2:A:412:HOH:O	2.60	0.48
1:A:126:ARG:HH22	1:A:178:GLN:HE21	1.60	0.48
1:A:162:GLU:O	1:A:166:ARG:HG3	2.14	0.48
1:A:128:MET:HE2	1:A:128:MET:HA	2.00	0.43
1:A:128:MET:CE	1:A:128:MET:HA	2.49	0.42

There are no symmetry-related clashes.

5.3 Torsion angles (i)

5.3.1 Protein backbone (i)

In the following table, the Percentiles column shows the percent Ramachandran outliers of the chain as a percentile score with respect to all X-ray entries followed by that with respect to entries of similar resolution.

The Analysed column shows the number of residues for which the backbone conformation was analysed, and the total number of residues.

Mol	Chain	Analysed	Favoured	Allowed	Outliers	Percentiles	
1	A	192/208 (92%)	189 (98%)	3 (2%)	0	100	100

There are no Ramachandran outliers to report.

5.3.2 Protein sidechains (i)

In the following table, the Percentiles column shows the percent sidechain outliers of the chain as a percentile score with respect to all X-ray entries followed by that with respect to entries of similar resolution.

The Analysed column shows the number of residues for which the sidechain conformation was analysed, and the total number of residues.

Mol	Chain	Analysed	Rotameric	Outliers	Percentiles
1	A	151/160 (94%)	148 (98%)	3 (2%)	58 34

All (3) residues with a non-rotameric sidechain are listed below:

Mol	Chain	Res	Type
1	A	31	LEU
1	A	88	LEU
1	A	156	GLU

Some sidechains can be flipped to improve hydrogen bonding and reduce clashes. All (1) such sidechains are listed below:

Mol	Chain	Res	Type
1	A	178	GLN

5.3.3 RNA (i)

There are no RNA molecules in this entry.

5.4 Non-standard residues in protein, DNA, RNA chains (i)

There are no non-standard protein/DNA/RNA residues in this entry.

5.5 Carbohydrates (i)

There are no carbohydrates in this entry.

5.6 Ligand geometry (i)

There are no ligands in this entry.

5.7 Other polymers (i)

There are no such residues in this entry.

5.8 Polymer linkage issues (i)

There are no chain breaks in this entry.

6 Fit of model and data (i)

6.1 Protein, DNA and RNA chains (i)

In the following table, the column labelled '#RSRZ> 2' contains the number (and percentage) of RSRZ outliers, followed by percent RSRZ outliers for the chain as percentile scores relative to all X-ray entries and entries of similar resolution. The OWAB column contains the minimum, median, 95th percentile and maximum values of the occupancy-weighted average B-factor per residue. The column labelled 'Q< 0.9' lists the number of (and percentage) of residues with an average occupancy less than 0.9.

Mol	Chain	Analysed	<RSRZ>	#RSRZ>2			OWAB(Å^2)	Q<0.9
1	A	198/208 (95%)	-0.08	4 (2%)	65	68	9, 18, 33, 59	1 (0%)

All (4) RSRZ outliers are listed below:

Mol	Chain	Res	Type	RSRZ
1	A	193	MET	4.5
1	A	65	GLY	3.3
1	A	156	GLU	2.6
1	A	159	GLN	2.3

6.2 Non-standard residues in protein, DNA, RNA chains (i)

There are no non-standard protein/DNA/RNA residues in this entry.

6.3 Carbohydrates (i)

There are no carbohydrates in this entry.

6.4 Ligands (i)

There are no ligands in this entry.

6.5 Other polymers (i)

There are no such residues in this entry.
